# Hsa_Circ_0000615-Encoded 250-Amino Acid Polypeptide Suppresses Epithelial Ovarian Cancer Progression via Promoting HMGB1 Nuclear Accumulation

**DOI:** 10.32604/or.2026.087931

**Published:** 2026-10-28

**Authors:** Ziyi Guo, Siyun Deng, Jiao Meng, Xiaofei Zhang, Yueyou Wang, Jiami Huang, Tonglu Li, Min Liu, Henghui Yang, Fangzhou Chen, Yuan Wang, Wei Meng, Shaojia Wang, Yanli Li

**Affiliations:** 1 Lab for Noncoding RNA & Cancer, School of Life Sciences, Shanghai University, Shanghai, China; 2 Department of Integration of Western and Traditional Medicine, Obstetrics and Gynecology Hospital of Fudan University, Shanghai, China; 3 Department of Gynecology, Shanghai Key Laboratory of Maternal Fetal Medicine, Shanghai Institute of Maternal-Fetal Medicine and Gynecologic Oncology, Shanghai First Maternity and Infant Hospital, School of Medicine, Tongji University, Shanghai, China; 4 Department of Pathology, Shuguang Hospital, Shanghai University of Traditional Chinese Medicine, Shanghai, China; 5 Gynecology Department of Traditional Chinese Medicine, The First Affiliated Hospital of Naval Medical University, Shanghai, China; 6 Department of Gynecology, The Third Affiliated Hospital of Kunming Medical University, Yunnan Cancer Hospital, Kunming, China; 7 Department of Pathology, Shanghai General Hospital, Shanghai Jiao Tong University School of Medicine, No.100 Haining Road, Shanghai, China

**Keywords:** Epithelial ovarian cancer, hsa_circ_0000615, ZNF609-250aa, high-mobility group box 1 (HMGB1), ubiquitination

## Abstract

**Objectives:** Circular RNAs (circRNAs) can encode functional peptides to regulate tumor progression, yet the roles of circRNA-encoded peptides in epithelial ovarian cancer (EOC) remain poorly understood. This study aimed to investigate the function of hsa_circ_0000615 (circ_0615) and its encoded polypeptide in EOC and explore its potential pre-clinical value as a candidate therapeutic target. **Methods:** We detected circ_0615 expression in EOC clinical tissues and cell lines. *In vitro* functional assays, *in vivo* xenograft and Patient-derived xenograft (PDX) models with adeno-associated virus (AAV)-mediated overexpression were used to assess tumor-suppressive phenotypes. Mass spectrometry, co-immunoprecipitation, western blotting, immunofluorescence, and chromatin immunoprecipitation were applied to characterize the circ_0615-encoded polypeptide Zinc Finger Protein 609-250-amino acid (ZNF609-250aa) and its interaction with high-mobility group box 1 (HMGB1). **Results:** circ_0615 was downregulated in EOC tissues and cell lines. Circ_0615 overexpression suppressed EOC proliferation and migration *in vitro* and *in vivo*. Mechanistically, circ_0615 promoted translation of ZNF609-250aa via eukaryotic translation initiation factor 2 subunit 1 (eIF2α). ZNF609-250aa bound HMGB1, reduced its ubiquitination, and promoted HMGB1 nuclear accumulation, which may contribute to repression of aldehyde dehydrogenase 1 family member A1 (ALDH1A1) transcription. AAV2/8-ZNF609-250aa restrained tumor growth in PDX models. **Conclusion:** Circ_0615-encoded ZNF609-250aa acts as a tumor suppressor in EOC, partially through modulating ZNF609-250aa-HMGB1-related molecular events. This study provides novel mechanistic insights and promising pre-clinical candidate targets requiring further validation for EOC intervention.

## 1 Introduction

Ovarian cancer (OC), predominantly presenting as epithelial ovarian cancer (EOC), is one of the gynecological malignancies with a five-year survival rate of merely 51% [1]. Owing to the lack of obvious early symptoms, the majority of OC patients are diagnosed at an advanced stage [2,3]. Debulking surgery in combination with platinum-taxane chemotherapy constitutes the first-line treatment strategy for OC [4,5], but faces challenges of poor prognosis and high recurrence rates caused by platinum resistance [6]. To some degree, the application of novel targeted therapies may overcome these challenges through specific regulation of oncogene expression. Currently, multiple targeted agents, including poly(ADP-ribose) polymerase inhibitors (Olaparib, Niraparib) and the anti-VEGF monoclonal antibody Bevacizumab, have been approved for OC treatment and have demonstrated clinical efficacy [7,8].

Circular RNA (circRNA) is a non-coding RNA (ncRNA) formed by covalently linking the two ends of a linear RNA. Its closed-loop structure confers exceptional stability within the cellular environment, with dual pro-tumor and anti-tumor properties in malignancies [9,10,11]. Benefiting from such structural characteristics, circRNAs can serve as ceRNAs for sponging microRNAs (miRNAs), or exert biological functions by encoding functional peptides dependent on internal ribosomal entry site (IRES) or N6-methyladenosine (m^6^A) modification [12,13,14]. Hsa_circ_0000615 (circ_0615, circZNF609), formed by the circularization of exon 2 of the zinc finger protein 609 (ZNF609) gene, has been reported to frequently act as a miRNA sponge regulating tumor progression in multiple cancers [15,16,17,18,19], and drive malignant progression in OC via the miR-324-5p/VDAC1 axis [20]. Zinc Finger Protein 609-derived 250-amino acid polypeptide (ZNF609-250aa) encoded by circZNF609 was initially found to be translated via the IRES region of circZNF609 and regulates muscle cell proliferation [21]. Additionally, the ZNF609-250aa induces apoptosis and ischemic acute kidney injury through the AKT/mTOR signaling pathway [22]. However, the roles of ZNF609-250aa in cancer remain poorly explored.

High mobility group box 1 (HMGB1) is a highly conserved non-histone chromatin-binding protein that undergoes nuclear-cytoplasmic translocation under specific stimuli such as inflammation, oxidative stress, or cellular injury, thereby performing different functions within the cell [23,24,25]. In the cytoplasm, HMGB1 participates in regulating the balance between autophagy and apoptosis and modulates immune responses [26,27,28,29]. Within the nucleus, HMGB1 stabilizes chromatin structure and regulates DNA repair and transcription [30,31,32]. However, the regulatory mechanisms governing HMGB1 nuclear translocation remain incompletely understood. Accumulating studies have identified multiple E3 ubiquitin ligases (CHIP, SYVN1, Parkin) that trigger ubiquitin-dependent proteasomal degradation of cytoplasmic HMGB1, whereas UCHL3 reverses this modification to maintain HMGB1 stability [33,34,35,36]. Notably, deubiquitination was observed to promote the stability and nuclear accumulation of certain proteins, such as p53 and SHMT1 [37,38], suggesting that ubiquitination may act similarly for HMGB1. Additionally, some studies have highlighted the role of HMGB1 in the malignant progression and drug resistance of OC [39,40], while the mechanisms underlying these effects due to localization require further research.

In this study, we aimed to explore the role and underlying molecular mechanism of the circ_0615-encoded polypeptide in EOC progression. We found that circ_0615 was significantly downregulated in EOC tissues according to circRNA sequencing (circRNA-Seq) data from normal ovarian and EOC tissues, and its tumor-suppressive effect against EOC was verified in *in vitro* and *in vivo*. Mass spectrometry confirmed that circ_0615 encodes the polypeptide ZNF609-250aa in EOC cells. Furthermore, circ_0615 binds to eukaryotic translation initiation factor 2 subunit 1 (eIF2α), and this interaction contributes to the production of this polypeptide. Mechanistic assays reveal that ZNF609-250aa interacts with HMGB1, inhibits ubiquitin-mediated degradation of HMGB1, and facilitates the nuclear localization of HMGB1. Chromatin immunoprecipitation (ChIP) assays further demonstrated that nuclear HMGB1 binds to the promoter region of the oncogene aldehyde dehydrogenase 1 family member A1 (ALDH1A1), represses ALDH1A1 transcription, and ultimately restrains OC progression. Additionally, adeno-associated virus (AAV) carrying ZNF609-250aa suppresses tumor growth in an EOC patient-derived xenograft (PDX) model. Collectively, these findings further suggest the potential functions of circRNAs and their encoded polypeptides for targeted therapies against EOC.

## 2 Materials and Methods

### 2.1 EOC Clinical Specimens

Eighty normal ovarian tissue samples and 70 EOC tissue samples were obtained from the Ethics Committee of Yunnan Cancer Hospital (YNCH). Normal ovarian tissues were obtained from patients with cervical cancer or uterine fibroids who underwent bilateral adnexectomy, and these ovarian tissues were pathologically confirmed to be histologically normal. All EOC specimens used for circRNA level detection were primary tumors. The majority of patients were pathologically diagnosed with high-grade serous ovarian carcinoma, while 6 cases corresponded to other epithelial ovarian carcinoma histological subtypes. The clinical tissue sample information is presented in Table S1. The ethical approval was granted by the Ethics Committee of Yunnan Cancer Hospital (Approval No.: KYCS2024-234). All procedures involving human participants were conducted in accordance with the principles of the Declaration of Helsinki. Written informed consent was obtained from all patients. Human tissue samples used in this study were fully de-identified to protect patient privacy.

### 2.2 CircRNA/mRNA Sequencing (circRNA/mRNA-Seq)

Eight normal ovarian tissues (designated Normal, N; from patients with cervical cancer or uterine fibroids who underwent bilateral adnexectomy and were pathologically confirmed to have normal ovaries) and 10 EOC tissues (designated Tumor, T) were collected with approval from the Ethics Committee of Yunnan Cancer Hospital. The circRNA-Seq library was first treated with RNase R to degrade linear RNA (EpiCentre, Inc., RNR07250, Middleton, Wisconsin, USA), and the strand-specific transcriptome library was constructed per the protocol of TruSeq^®^ Stranded Total RNA Library Prep Plant (Illumina, RS-122-2001, San Diego, CA, USA) using 2 μg of total RNA.

Sequencing data processing, circRNA identification/annotation, and differential expression statistics were performed by Shanghai Majorbio Biopharm Technology Co., Ltd. Low-quality reads and adapters were removed prior to analysis. Genes were then excluded if (i) they had zero read counts in more than 50% of samples within either group, (ii) their gene symbols were missing or could not be matched to an annotated circRNA, or (iii) their mean read counts were extremely low (<20) in both groups despite apparent differences. The company supplied the raw count matrix, group mean expression levels, *t*-test *p*-values, and Fold change (FC). *p*-values of the remaining genes were adjusted for multiple testing using the false discovery rate (FDR) method (Benjamini-Hochberg) in Microsoft Excel. Differentially expressed circRNAs (DECs) were defined as |log_2_FC| > 0.5 (T vs. N) combined with FDR-adjusted *p* < 0.05. Genes were ranked by ascending FDR-adjusted *p*, and the top 20 most significantly altered circRNAs were selected. Row-wise Z-score normalization was manually performed in Excel for heatmap plotting, and the heatmap together with the volcano plot was plotted by GraphPad Prism 10 (GraphPad Software, Inc., Boston, MA, USA).

For mRNA-Seq library construction, the RNA-seq transcriptome library was prepared following the SMART-Seq_V4 Ultra Low Input RNA Kit for Sequencing (Clontech, 634888, Mountain View, CA, USA) using 10 ng of total RNA. Three biological replicates were included per group, consisting of 3 Hey NC samples and 3 Hey ZNF609-250aa-Flag overexpression samples. Strand-specific libraries were constructed via dUTP incorporation during second-strand cDNA synthesis. Library quality was assessed by Qubit^®^ 2.0 Fluorometer (Life Technologies, Carlsbad, CA, USA), qPCR and Agilent 2100 Bioanalyzer system (Agilent Technologies, Santa Clara, CA, USA). Raw reads were filtered by fastp (v0.23.2, HaploX Biotechnology, Shenzhen, China) to obtain clean reads. Clean reads were aligned to the human reference genome with HISAT2 (v2.2.1, Johns Hopkins University, Baltimore, MD, USA). Gene expression was quantified by featureCounts (v2.0.6, Walter and Eliza Hall Institute of Medical Research, Parkville, VIC, Australia) to calculate FPKM values. Differential expression analysis was performed using the DESeq2 R package (v1.44.0, Fred Hutchinson Cancer Research Center, Seattle, WA, USA), with the threshold defined as adjusted *p* value (*padj*) < 0.05 and |log_2_(FC)| > 1). All detection steps were performed on an Illumina NovaSeq™ X Plus sequencer (Illumina, San Diego, CA, USA), and the sequencing service was provided by Novogene Co., Ltd. (Novogene Co., Ltd., Beijing, China).

### 2.3 Cell Lines and Cell Culture

The human normal ovarian epithelial cell line IOSE-80 (RRID: CVCL_5546; Cat. No. H6-1901) was obtained from OriCell (Cyagen Biosciences, Suzhou, China). Human EOC cell lines Skov3 (RRID: CVCL_0532; Cat. No. CL-0215), Hey (RRID: CVCL_0297; Cat. No. CL-0671), A2780 (RRID: CVCL_0134; Cat. No. CL-0013), OVCAR3 (RRID: CVCL_0465; Cat. No. CL-0178), OVCAR-8 (RRID: CVCL_1629; Cat. No. CL-0759), HO8910 (RRID: CVCL_6868; Cat. No. CL-0113) and human primary embryonal kidney cell line 293T (HEK-293T; RRID: CVCL_0063; Cat. No. CL-0005) were purchased from Procell Life Science & Technology Co., Ltd. (Wuhan, China). Human EOC cell line TOV-112D (RRID: CVCL_3612; Cat. No. CRL-11731) was obtained from the American Type Culture Collection (ATCC, Manassas, VA, USA). All cell lines were authenticated by STR profiling and confirmed to be negative for mycoplasma. The Skov3 and OVCAR3 cell lines were cultured in RPMI-1640 medium (HyClone, SH30809.01, Logan, UT, USA), and the other cell lines were cultured in Dulbecco’s modified Eagle’s medium (DMEM, HyClone, SH30022.LS). All media were supplemented with 10% fetal bovine serum (FBS, Gibco, 10099141, Gaithersburg, MD, USA) and an antibiotic cocktail of 1% penicillin and streptomycin (HyClone, SV30010). All the cells were maintained at 37°C under 5% CO_2_.

### 2.4 Plasmid Construction and Cell Transfection

Circ_0615, circ_0615-Flag, and circ_0615-mut (pLenti ciR6024 CMV-MCS-EF1A-luciferase-T2A-puro) were constructed by BioLinker (Shanghai, China), and circ_NC served as the control. For the circ_0615-mut plasmid, on the backbone of the circ_0615-Flag vector, the start codon ATG inside the ORF of circ_0615 was point-mutated to ACG to eliminate translation initiation of ZNF609-250aa without inducing a frameshift mutation of downstream sequences. The pLKO.1-TRC-circ_0615-sh (circ_0615-sh) was constructed for circ_0615 knockdown, and pCDH-CMV-MCS-EF1A-Puro-ZNF609-250aa-Flag (ZNF609-250aa-Flag) was constructed to overexpress ZNF609-250aa with Flag tags, while circ_shNC and pCDH-CMV-MCS-EF1A-Puro empty vector (NC) served as the corresponding controls. Circ_0615, circ_0615-sh, circ_0615-Flag, circ_0615-mut, ZNF609-250aa-Flag stable-overexpressing EOC cell lines (Hey and Skov3) and their corresponding controls were established by lentiviral transduction in this study. The overexpression and knockdown efficiency and specificity of circ_0615 in stable cell lines was verified by divergent primer-based RNA extraction and quantitative real-time polymerase chain reaction (qRT-PCR), which distinguishes the circular circ_0615 transcript from its linear host transcript. For stable expression, the above plasmids were co-transfected with packaging vectors psPAX2 and pMD2.G at a 4:3:1 ratio into HEK293T cells. Lentivirus production was performed using Lipofectamine 3000 (Invitrogen, L3000015, Carlsbad, CA, USA) following the manufacturer’s protocol. Cell supernatants were collected at 48 and 72 h post-transfection, passed through a 0.45 μm filter. Lentiviral transduction was performed on EOC cells at 60–70% confluence with a MOI of 10, supplemented with polybrene (8 μg mL^−1^). Twenty-four hours after lentiviral transduction, when cell confluence reached 30–50%, puromycin (2 μg mL^−1^) was added for 48 h for positive cell selection. Successful establishment of stable cell lines was confirmed by qRT-PCR and western blot assays to detect the expression level of target circRNA or protein.

SiRNAs of eIF2α and HMGB1, and corresponding control siRNA (si-NC) were synthesized by OBiO Technology (Guangzhou, China) and were transiently transfected into EOC cells at a final concentration of 50 nM using Lipofectamine 2000 (Invitrogen, 11668027) when cells reached 50% confluence. Forty-eight hours post-transfection, cellular RNA or protein was obtained for subsequent analysis. Knockdown efficiencies of eIF2α and HMGB1 were evaluated by qRT-PCR at the RNA level before functional experiments, and corresponding results were presented in the Section 3. All procedures were strictly conducted in accordance with the manufacturer’s protocols. The sequences of all the siRNAs and shRNAs are listed in Table S2.

### 2.5 Cell Proliferation Assay and Colony Formation Assay

Cell proliferation was determined with CCK-8 assay (Yeasen Biotech, 40203ES60, Shanghai, China). Briefly, EOC cells were seeded into a 96-well plate (LABSELECT, 11510, China) at a density of 1.5 × 10^3^ Hey cells and 2.0 × 10^3^ Skov3 cells per well. A 5% CCK-8 working solution was prepared in basal medium according to the manufacturer’s instructions, and added to each well at 0, 24, 48 and 72 h and incubated for 2 h at 37°C with 5% CO_2_. Absorbance was detected at 450 nm using a Multiskan FC microplate reader (Thermo Fisher Scientific, USA). Blank wells containing only basal medium (without cells) were included for background subtraction alongside experimental and control wells at each time point; an equal volume of 5% CCK-8 working solution was added to blank wells and measured synchronously. All absorbance values used for analysis were blank-corrected. Each group contained four technical replicate wells per time point, and the assay was independently repeated three times.

For the colony formation assay, 800 EOC cells were seeded into 6-well plates (LABSELECT, 11110). After 12–14 days of incubation at 37°C with 5% CO_2_, the cell colonies were first fixed using methanol at room temperature for 15 min, and then stained with crystal violet (0.5% w/v) at room temperature for 30 min. The colonies were photographed and counted.

### 2.6 Cell Migration Assay

For the wound healing assay, Hey and Skov3 cells were plated into a 6-well plate at a seeding density of 5 × 10^5^ cells per well until the cell confluence reached 100%. Cells were pretreated with Mitomycin C (10 μg mL^−1^) for 2 h to inhibit cell proliferation. After pretreatment, the medium was aspirated, and cells were rinsed twice with PBS to remove Mitomycin C. A single-scratch wound was made in the middle of each well with a 200 μL sterile pipette tip. Subsequently, the cells were cultured in FBS-free basal medium for 24 h. Cell migration images at 0 h and 24 h were captured using an Olympus CX21FS1 optical microscope (Olympus, Tokyo, Japan) equipped with ToupView v4.1 (Hangzhou ToupTek Photonics Co., Ltd.). Wound closure was quantified by measuring wound area at 0 h and the indicated time-points using ImageJ (version 1.54g, National Institutes of Health, USA). The percentage of wound closure was calculated with the following formula: percentage of wound closure (%) = [(Initial wound area − Wound area at indicated time point)/Initial wound area] × 100%.

For the Transwell assay, a total of 1 × 10^5^ EOC cells were resuspended in 200 μL of FBS-free basal medium and seeded into Transwell chambers (8.0 μm pore size; Corning, Inc., 3422, New York, NY, USA) in a 24-well culture plate (LABSELECT, 11310). The lower chamber was filled with 800 μL of complete medium containing 20% FBS to form a chemotactic gradient. All cells were cultured under routine experimental conditions of 37°C and 5% CO_2_. After a fixed incubation duration of 48 h, the non-migrated cells on the upper surface of the Transwell membrane were gently wiped off with a sterile cotton swab. The migrated cells on the lower membrane surface were fixed with methanol for 15 min and stained with crystal violet (0.5% w/v) for 30 min. Cell migration was quantified by capturing five randomly selected microscopic fields per Transwell insert at 10× magnification, and the number of migrated cells was counted automatically using ImageJ (v1.54f, NIH). Both wound-healing and Transwell migration assays were performed with three technical replicates per group and repeated in three independent biological experiments.

### 2.7 Cell Apoptosis Assay

A total of 1 × 10^5^ EOC cells after plasmid transfection were plated in each well of a six-well plate until the density reached 90%. For apoptosis induction, cells were exposed to 253.7 nm ultraviolet light at a distance of 20 cm for 2 h to induce DNA-damage-related apoptotic stress. The cells were collected, washed, and stained with BD Pharmingen™ FITC Annexin V Apoptosis Detection Kit I (BD Biosciences, 556574, New York, NY, USA) in accordance with the manufacturer’s instructions. Briefly, cells were resuspended in binding buffer, followed by addition of FITC-Annexin V and PI reagents, and incubated for 15 min at room temperature in the dark. Samples were filtered through a 50 μm filter membrane prior to acquisition. Experiments were performed using a MoFlo XDP flow cytometer sorting system (Beckman Coulter, Brea, CA, USA). Unstained control and single-stained control tubes (Annexin-V-only and PI-only) were prepared for fluorescence compensation and quadrant gate setup. Cell debris and cell doublets were excluded during gating; a minimum of 10 000 events were acquired for each sample. Viable cells were defined as Annexin V-FITC^-^/PI^-^, early-apoptotic cells as Annexin V-FITC^+^/PI^-^, late-apoptotic cells as Annexin V-FITC^+^/PI^+^, and necrotic cells as Annexin V-FITC^-^/PI^+^. The total apoptotic rate was calculated as the sum of early- and late-apoptotic cell proportions. This assay was carried out with three technical replicates per group across three independent biological experiments.

### 2.8 Protein Extraction and Western Blot Analysis

Total protein was extracted from the cells using RIPA lysis buffer with a protease inhibitor cocktail (Leagene Biotechnology, PS0009, Beijing, China), and quantified with a Protein BCA Assay Kit (Yeasen Biotech, 20201ES76) as previously described [41]. Briefly, the protein was denatured in 1× SDS loading buffer (containing 2% SDS, 100 mM DTT as a reducing agent) at 100°C for 10 min. Protein samples were separated by sodium dodecyl sulfate polyacrylamide gel electrophoresis (SDS-PAGE) using 10%~12.5% separating gel; electrophoresis was performed at 80 V for the stacking gel and 120 V for separating gel, transferred to a polyvinylidene difluoride (PVDF) membrane following sodium dodecyl sulfate polyacrylamide gel electrophoresis (SDS-PAGE) and then blocked in 5% nonfat powdered milk for 1.5 h at room temperature followed by incubation at 4°C overnight with primary antibodies. Antibodies against the following proteins were used: anti-ubiquitin (Cell Signaling Technology, 3933S, Boston, MA, USA, 1:1000), anti-eIF2α (Santa Cruz Biotechnology, sc-133132, USA, 1:1000), anti-ZNF609 (Thermo Fisher Scientific, PA5-59314, Boston, MA, USA, 1:500), anti-HMGB1 (Proteintech, 10829-1-AP, Wuhan, China, 1:2000), anti-ALDH1A1 (Proteintech, 15910-1-AP, 1:2000), anti-Flag (DYKDDDDK) (Proteintech, 66008-4-Ig, 1:2000), anti-HA (Proteintech, 51064-2-Ig, 1:2000), anti-Lamin B1 (Proteintech, 12987-1-AP, 1:1000), anti-GAPDH (Proteintech, 10494-1-AP, 1:5000) and anti-β-Tubulin (Proteintech, 10094-1-AP, 1:5000). The PVDF membrane was washed with TBST buffer 3 times and incubated with HRP-conjugated goat anti-rabbit secondary antibody (Beyotime, A0208, Shanghai, China, 1:3000) or HRP-conjugated goat anti-mouse secondary antibody (ABclonal, AS003, Wuhan, China, 1:3000) for 1.5 h at room temperature. Finally, protein bands were visualized with a chemiluminescent HRP substrate (ABclonal, RM02867) and exposed using an E-Gel Imager (Tanon, Shanghai, China). ImageJ (v1.54f, NIH) was used for grayscale analysis. Band intensities of target proteins were normalized to the corresponding loading controls (β-Tubulin for total protein, GAPDH for cytoplasmic fraction, Lamin B1 for nuclear fraction), and relative protein expression was calculated as the ratio of target band intensity to loading-control band intensity. All relative expression values of experimental groups were further normalized to those of the control group for comparative analysis. In protein stability analysis, cells were treated with 100 μg mL^−1^ cycloheximide (CHX) (MedChemExpress, HY-12320, Monmouth Junction, NJ, USA) for 0, 6, 12 and 18 h before protein extraction, or treated with 20 μM MG-132 or 20 μM chloroquine (CQ) (MedChemExpress, HY-17589A) for 8 h. All western blot experiments were performed in three independent biological replicates, and quantitative results were derived from these independent experiments.

### 2.9 RNA Extraction and Quantitative Real-Time Polymerase Chain Reaction (qRT-PCR) Analysis

Total RNA of tissues and cells was extracted by TRIzol UP (TransGen Biotech, ET111, Beijing, China). A total of 1 μg RNA was used as input for reverse-transcription. Reverse-transcription was divided into gDNA-removal and cDNA-synthesis steps; the gDNA-removal reaction was performed in a 10 μL system at 42°C for 2 min, followed by cDNA synthesis in a total volume of 20 μL at 37°C for 30 min and 85°C for 5 min. Reverse transcription was performed with the PrimeScript RT reagent Kit with gDNA Eraser Kit (TaKaRa Biotech, RR047A, Otsu, Japan) following the manufacturer’s instructions. The primers were designed to cover the junction sites for circ_0615. RNA levels were quantified by qRT-PCR using Hieff qPCR SYBR Green Master Mix (Yeasen Biotech, 11201ES08). The qRT-PCR was carried out in a 10 μL total reaction system containing 2 μL of cDNA template (10 ng/μL), 0.4 μL of forward primer (10 μM), 0.4 μL of reverse primer (10 μM), 5 μL of Hieff qPCR SYBR Green Master Mix, and RNase-free ddH_2_O. Cycling parameters were 95°C for 3 min, followed by 40 cycles of 95°C for 30 s, gene-specific annealing temperature for 25 s, and 72°C for 30 s. Melting-curve analysis was applied to verify amplification specificity. 18S RNA as the endogenous control. The relative quantification (2^−ΔΔCt^) method was used to analyze the data. Primer sequences and annealing temperatures are listed in Table S2. All qRT-PCR experiments were performed with three independent biological replicates and three technical replicates for each sample.

### 2.10 RNase R Treatment

A total of 1 μg RNA samples were digested with or without 6 U/μg RNase R (Beyotime, R7092) in a final reaction volume of 20 μL containing 10× RNase R Reaction Buffer, and RNase-free ddH_2_O, at 37°C for 90 min. Digested RNA was directly reverse-transcribed without further purification. Genomic DNA (gDNA) and corresponding cDNA templates were amplified using divergent and convergent primer pairs, respectively. Convergent primers amplify target fragments from both gDNA and cDNA templates, whereas divergent primers only generate amplicons from circRNA-derived cDNA and yield no product from gDNA, which was used to verify the back-splicing junction of circ_0615. The PCR amplification was performed in a 10 μL total reaction system, containing 1 μL of gDNA or cDNA template, 5 μL of 2× Hieff PCR Master Mix (Yeasen Biotech, 10136ES08), 0.4 μL forward primer (10 μM), 0.4 μL reverse primer (10 μM), and RNase-free ddH_2_O. Cycling parameters were as follows: initial denaturation at 95°C for 3 min, followed by 30 cycles of 95°C for 15 s, optimal annealing temperature for 35 s, and 72°C for 15 s; a final extension step was carried out at 72°C for 5 min. The products of PCR were analyzed via 1% agarose gel electrophoresis. Primer sequences are described in Table S2.

### 2.11 Actinomycin D Assay

EOC cells were seeded in a 6-well plate and treated with 2 μg mL^−1^ actinomycin D (Act D) (MedChemExpress, HY-17559) at a density of 70%-80%. Total RNA was collected at 0, 6, 12, 18, 24 h after Act D treatment. qRT-PCR was performed to determine the relative RNA abundance of circ_0615 and linear ZNF609 mRNA. Primers for linear ZNF609 mRNA target exon 3, a genomic region not covered by circ_0615, ensuring specific amplification of linear ZNF609 mRNA without detecting the circ_0615 transcript. 18S rRNA was used as the reference gene for qRT-PCR normalization. Three independent biological replicates and three technical replicates were performed for each time-point. Relative RNA abundance at each time-point was normalized to the 0 h Act D-treated group.

### 2.12 Cellular Fractionation

The Ambion PARIS Kit (Thermo Fisher Scientific, AM1921) was used for isolation of subcellular fractions according to the manufacturer’s instructions. For each biological replicate, approximately 2 × 10^7^ cells were pooled and resuspended in pre-chilled Cell Fractionation Buffer, followed by 5 min incubation on ice. Cytoplasmic and nuclear fractions were separated by centrifugation at 500× *g* for 5 min at 4°C. The resulting cytoplasmic supernatant and nuclear pellet were aliquoted equally, and aliquots from the same set of fractionated samples were separately used for subsequent RNA and protein extraction. RNA of Hey and Skov3 cells was extracted from the cytoplasmic and nuclear fractions and subjected to qRT-PCR, while GAPDH mRNA was used as a cytoplasmic marker, and U6 snRNA was used as a nuclear marker. Protein extraction was simultaneously conducted to verify the purification quality and detected by western blot. GAPDH and Lamin B1 were applied as cytoplasmic and nuclear fraction markers rather than general endogenous loading controls. Three independent biological replicates were performed for this assay.

### 2.13 RNA Fluorescence In Situ Hybridization (FISH)

Specific Cy3-labeled FISH probes against circ_0615 were designed and synthesized by GenePharma (Shanghai, China) to target nucleotides 789-803 of the circ_0615 transcript, and the assay was performed using the Fluorescent *In Situ* Hybridization Kit (GenePharma, Shanghai) following the manufacturer’s instructions. Briefly, EOC cells were seeded on coverslips in 24-well plates and grown to 70%–80% confluence. After 1× PBS washing, cells were fixed in 4% paraformaldehyde and permeabilized using 0.5% Triton X-100. Pre-hybridization was subsequently carried out at 37°C. After denaturation, the probe working solution was applied to the cells for overnight hybridization under dark conditions. Sequential post-hybridization washes were performed using pre-warmed washing solutions according to the kit protocol. Finally, coverslips were mounted with DAPI-containing anti-fade mounting medium. The fluorescence signal of stained cells was measured and photographed using an Olympus FV3000 confocal fluorescence microscope (Olympus) for preliminary observation of the subcellular localization of circ_0615. For each sample, at least five random visual fields were captured under Olympus FV3000 microscope (Olympus). The probe used for FISH is listed in Table S2.

### 2.14 Immunofluorescence (IF)

The experiment was performed as previously described [42]. Briefly, EOC cells were seeded at 5 × 10^4^ cells per well into 24-well plates containing round coverslips and fixed when reaching 30%–50% confluence. Cells were fixed in methanol for 15 min, followed by permeabilization with 0.5% Triton X-100 (Servicebio, GC204003, Wuhan, China) at 4°C for 20 min, then blocked in 5% BSA (Sangon Biotech, A600332, Shanghai, China) at 4°C overnight. For staining, coverslip samples were incubated with either single primary antibody or a combination of two primary antibodies: anti-Flag (Proteintech, 66008-4-Ig, host: mouse, 1:200) and anti-HMGB1 (Proteintech, 10829-1-AP, host: rabbit, 1:200) at 4°C overnight. After washing with phosphate-buffered saline Tween 20 (PBST), samples were incubated with corresponding secondary antibodies at room temperature for 1.5 h. Finally, coverslips were mounted with DAPI Fluoromount-G (SouthernBiotech, 0100-20, Birmingham, AL, USA). For single-staining of HMGB1, Alexa Fluor 488 AffiniPure goat anti-rabbit IgG (Jackson ImmunoResearch, 111-545-003, 1:200, West Grove, PA, USA) was used; for double-immunofluorescence staining, Alexa Fluor 488 AffiniPure goat anti-mouse IgG (Jackson ImmunoResearch, 115-545-003, 1:200) was applied to detect mouse-derived anti-Flag, whereas Alexa Fluor 594 AffiniPure goat anti-rabbit IgG (Jackson ImmunoResearch, 111-585-003, 1:200) was used for rabbit-derived anti-HMGB1. All stained cells were examined and photographed using a Zeiss LSM880 confocal fluorescence microscope (Carl Zeiss, Jena, Germany).

### 2.15 In Situ Proximity Ligation Assay (PLA)

The PLA was performed using a Duolink PLA *In Situ* Kit (Sigma-Aldrich, DU92101, Saint Louis, MO, USA) following the manufacturer’s instructions. EOC cells were seeded into 24-well plates containing round coverslips and were fixed and permeabilized as described for the IF. Anti-Flag (Proteintech, 66008-4-Ig, host: mouse, 1:200) was incubated with MINUS PLA probe, while anti-HMGB1 (Proteintech, 10829-1-AP, host: rabbit, 1:200) was incubated with PLUS PLA probe. The PLA signal was visualized using an Olympus FV3000 confocal fluorescence microscope (Olympus). All PLA experiments were performed in three independent biological replicates, with at least five random visual fields captured for each sample.

### 2.16 RNA Pull-down and Mass Spectra Analysis

The interaction between circ_0615 and proteins was detected with the Pierce Magnetic RNA-Protein Pull-Down Kit (Thermo Fisher Scientific, 20164Y) according to the manufacturer’s protocols. Biotin-labeled probes targeting the junction site of circ_0615 were synthesized by GenePharma, and circ_NC negative control oligo probe was used as a negative control. Briefly, the cells were lysed by RIPA lysis buffer (Leagene Biotechnology, Beijing, P1005). The cell lysate was incubated with a biotin-labeled circ_0615 probe together with streptavidin magnetic beads. Captured interacting proteins were visualized by silver staining using the Fast Silver Stain Kit (Beyotime, P0017S). Mass-spectrometry identification was performed by BioTREE Biomedical Technology (Shanghai, China) based on proteins enriched from pull-down products.

### 2.17 RNA Binding Protein Immunoprecipitation Assay (RIP)

The RIP assay was performed using a Magna RIP Kit (Millipore Corporation, 17-700, Billerica, MA, USA). Briefly, approximately 1 × 10^7^ EOC cells were used for each individual RIP reaction. Cells were scraped, collected, and lysed in 120 μL kit-supplied RIP lysis buffer supplemented with 0.6 μL protease inhibitor cocktail and 0.3 μL RNase inhibitor before storage at −80°C overnight. 5 μg anti-eIF2α (Santa Cruz Biotechnology, host: mouse, sc-133132) or 5 μg Normal Mouse IgG (Proteintech, B900620) was pre-coated onto 50 μL protein A/G magnetic beads supplied within the Magna RIP Kit, and incubated for 1 h before incubating with cell lysate at 4°C overnight. The beads were washed 6 times with kit-provided RIP Wash Buffer at room temperature using a magnetic separator, followed by protein digestion and RNA extraction. The cDNA library of circRNAs was constructed using the PrimeScript RT reagent Kit with gDNA Eraser (TaKaRa Biotech, RR047A), and the RNA level of circ_0615 was measured by qRT-PCR, while the protein levels of eIF2α were detected by western blot.

### 2.18 Co-Immunoprecipitation Assay and Mass Spectrometry Analysis (Co-IP)

For each independent Co-IP reaction, approximately 4 × 10^6^ EOC cells were lysed in 150 μL IP lysis buffer (Leagene Biotechnology, P1005) supplemented with 1.5 μL PMSF. The Co-IP assay was performed using Protein A/G Immunoprecipitation MagBeads (Bimake, B23202, Shanghai, China) following the manufacturer’s instructions. Rabbit IgG control Polyclonal antibody (Proteintech, 30000-0-AP, 2 μg), Normal Mouse IgG (Proteintech, B900620), anti-Flag (Proteintech, 66008-4-Ig, host: mouse, 2 μg), anti-HMGB1 (Proteintech, 10829-1-AP, host: rabbit, 2 μg) were incubated with Magbeads used for immunoprecipitation. Anti-Flag, anti-HMGB1, anti-HA (Proteintech, 51064-2-Ig, 2 μg), and anti-ubiquitin (Cell Signaling Technology, 3933S, 2 μg) were applied for subsequent western blot detection. Input lysate and species-matched IgG negative control were included in Co-IP and processed in parallel with immunoprecipitation samples. Protein samples were separated on 10% SDS-PAGE gels, followed by silver staining using the Fast Silver Stain Kit (Beyotime, P0017S). For silver staining, gels were briefly rinsed with ultrapure water after electrophoresis, fixed overnight at temperature, washed with 30% ethanol and ultrapure water sequentially, incubated with sensitization solution, silver staining solution, and developed until distinct bands appeared; the reaction was terminated with stop solution, followed by final ultrapure-water washing and imaging under white-light illumination. Specific bands distinguished from the IgG-negative lane were excised, immersed in ddH_2_O, and sent to Shanghai Biotree Biomedical Technology (Shanghai, China) for mass spectral analysis.

### 2.19 Chromatin Immunoprecipitation (ChIP)

The Pierce Magnetic ChIP Kit (Thermo Fisher Scientific, 26157) was used to perform the ChIP according to the manufacturer’s protocol. Briefly, cells were chemically crosslinked with 1% formaldehyde for 10 min at room temperature, followed by incubation with 2.5 M glycine (final concentration of 125 mM) for 5 min to quench cross-linking and preserve DNA-protein interactions. Cells were harvested and lysed with membrane extraction buffer supplemented with protease and phosphatase inhibitors. Nuclei were resuspended in MNase buffer and digested with MNase at 37°C for 15 min, followed by stop solution to terminate digestion. Nuclear membranes were disrupted by sonication on ice with 30 W power, five cycles of 45 s each (15 s on/30 s off) to obtain chromatin fragments. For immunoprecipitation, Rabbit IgG control Polyclonal antibody (Proteintech, 30000-0-AP, 2 μg) and anti-HMGB1 (Proteintech, 10829-1-AP, host: rabbit, 2 μg) were applied. Ten percent of chromatin lysate was reserved as an input control. DNA/protein complexes were captured by Protein A/G magnetic beads. After sequential washing, immune complexes were eluted, and cross-linking was reversed in the presence of NaCl and proteinase K. DNA fragments were purified with spin columns.

ChIP-seq library preparation and high-throughput sequencing were completed by Shanghai Like Biotechnology Co., Ltd. Briefly, 10 ng of purified ChIP DNA was used for library construction, including end repair, A-tailing, paired-end adapter ligation, PCR amplification and fragment purification. Valid libraries were sequenced on the Illumina HiSeq platform (Illumina Inc., San Diego, CA, USA) with paired-end sequencing mode. Raw sequencing reads were strictly quality-filtered to remove unreliable sequences. The filtering criteria excluded adapter-contaminated reads, reads with over 40% low-quality bases (Qphred ≤ 20), and sequences containing more than 5% ambiguous N bases. Clean reads were mapped to the reference genome using Bowtie2 (v2.2.5, Johns Hopkins University). Picard (v3.0.0, Broad Institute, Cambridge, MA, USA) was applied to remove PCR duplicates and filter low-quality and multi-mapped reads. Peak calling was performed by MACS2 (v2.2.6, Liu Lab, Cold Spring Harbor Laboratory, NY, USA) in narrow peak mode with q value < 0.05. Differential peak analysis was performed using DESeq2 (v1.44.0, Fred Hutchinson Cancer Research Center) and edgeR (v4.2.2, Bioconductor, Melbourne, VIC, Australia), with the screening criteria of |log_2_FC| ≥ log_2_(1.5) and *padj* < 0.05, or *p* < 0.05 for datasets with insufficient differential peaks. Integrative Genomics Viewer (IGV) software (v2.19.0, Broad Institute, Cambridge, MA, USA) was used for genome-wide visualization of peaks and read alignments.

ChIP-qPCR was performed to measure DNA enrichment, and results were calculated as fold-enrichment relative to control IgG. Three independent biological replicates were used for ChIP-qPCR and ChIP-seq, including 3 Hey NC samples and 3 Hey ZNF609-250aa-Flag overexpression samples. The probes used for qRT-PCR are listed in Table S2.

### 2.20 Subcutaneous Tumor Xenograft Assay

Five-week-old female nude BALB/c mice (body weight: 14–16 g) were obtained from Gempharmatech Co., Ltd. (Nanjing, China), with a total of 12 mice initially purchased. Animals were housed and operated in the specific-pathogen-free (SPF)-grade Laboratory Animal Center, School of Life Sciences, Shanghai University, with a 12 h light/dark cycle, constant temperature (22 ± 2°C) and humidity (50–60%), and fed with standard laboratory chow and sterile drinking water. One-week acclimatization was allowed prior to any experimental manipulation. Animals were randomly assigned to 2 groups (6 mice per group) before tumor-cell inoculation, and subsequently individually numbered via toe clipping under brief isoflurane anaesthesia. Each nude mouse was injected subcutaneously in the right flank with 5 × 10^6^ Hey cells stably transfected with circ_NC or circ_0615 (resuspended in 100 μL PBS). Mice that failed to develop detectable subcutaneous tumors or exhibited poor physical condition after cell injection were excluded from further analysis. The final number of evaluable mice was 5 per group. Sample size was determined based on empirical experience from previous subcutaneous xenograft studies. Tumor size was measured with calipers every 7 days, and the following formula was used to calculate tumor volumes: volume = length × width^2^/2. Pre-defined humane endpoint criteria included maximum tumor diameter <1.5 cm, body weight loss exceeding 20%, tumor ulceration, obvious mental distress, and impaired locomotor activity. Any mouse meeting one or more humane endpoint criteria would be humanely euthanized. Mice were sacrificed 5 weeks post-injection, and the xenograft tumors were excised and weighed. All animal experiments were carried out in accordance with the Institutional Animal Care and Use Committee of Shanghai University (Approval No.: YS2024-151). All efforts were made to minimize suffering, and experimental animals were humanely euthanized by well-trained researchers via cervical dislocation, which strictly complied with the American Veterinary Medical Association (AVMA) guidelines for animal euthanasia. Statistical analyses for tumor-related data were performed as described in Section 2.24. All animal procedures in this study were reported in accordance with the ARRIVE Essential 10 guidelines.

### 2.21 Establishment of Patient-Derived Xenograft (PDX) Model and AAV Therapy

Five-week-old female nude BALB/c mice (body weight: 14–16 g) were obtained from Gempharmatech Co., Ltd. (Nanjing, China), with a total of 10 mice initially purchased for the establishment of P0-generation PDX models. Animal housing, husbandry conditions, one-week acclimatization, and individual mouse identification via toe clipping under brief isoflurane anaesthesia were performed as described in Section 2.20. EOC patient-derived tissues were obtained from Yunnan Cancer Hospital. Fresh tumor tissues were immediately subcutaneously inoculated into both flanks of nude mice, which were defined as the P0 generation. An additional 10 five-week-old female nude BALB/c mice were purchased for P1-generation propagation. After the P0 models were successfully established, tumor-bearing P0 tissues were sectioned into 0.2 cm × 0.2 cm specimens, and transplanted into the bilateral flanks of five-week-old female nude mice to establish the P1 generation. Mice that failed to form palpable tumors after tissue transplantation, exhibited only unilateral tumor growth, or showed poor physical condition were excluded during PDX model propagation. Three weeks after transplantation, once tumors formed, six P1-generation mice were randomly selected for subsequent experiments and individually numbered via toe clipping.

AAV2/8-NC or AAV2/8-ZNF609-250aa (containing a Flag tag and a GFP reporter) were constructed by OBiO Technology (Guangzhou, China). AAV2/8-ZNF609-250aa virions were used for intraperitoneal ZNF609-250aa overexpression treatment, with AAV2/8-NC serving as the negative control. Treatment with AAV2/8-NC and AAV2/8-ZNF609-250aa was performed on bilateral tumors within the same mouse. Intraperitoneal injections of 1 × 10^11^ VP mL^−1^ virions of AAV2/8-NC or AAV2/8-ZNF609-250aa (100 μL per tumor) were administered once daily for seven consecutive days. Tumor size was measured with calipers every week, using the following formula: volume = length × width^2^/2. Pre-defined humane endpoint criteria were applied as described in Section 2.20. Four weeks after AAV administration, AAV-mediated GFP signalling within the tumor was observed via the IVIS imaging system. Mice were humanely euthanized 6 weeks post-AAV injection, and the xenograft tumors were excised and weighed. Statistical analyses for PDX-related tumor data were performed as described in Section 2.24.

### 2.22 Immunohistochemistry (IHC)

Tumor tissues from subcutaneous tumor xenograft and PDX models in mice were treated using tissue 10% fixative solution. All IHC staining procedures were performed by Servicebio (Wuhan, China). Briefly, primary tumor tissues were embedded in paraffin and subsequently cut into 4 μm-thick sections. The sections were deparaffinized, dehydrated through xylene and graded alcohols, and rehydrated with demineralized water. After being antigenically restored for 5 min and incubated in 3% H_2_O_2_ solution for 25 min at room temperature in the dark, the sections were incubated overnight at 4°C with primary antibodies: anti-Ki67 (Servicebio, GB111141, host: rabbit, 1:200), anti-Flag (Proteintech, 66008-4-Ig, host: mouse, 1:50), anti-HMGB1 (Proteintech, 10829-1-AP, host: rabbit, 1:50). After washing, sections were incubated with S-vision polymer HRP-conjugated anti-mouse/rabbit IgG secondary antibody (Servicebio, G1303) for 20 min at room temperature in a humidified chamber. Antibody binding signals were visualized using 3,3′-diaminobenzidine (DAB) chromogenic reagent (Servicebio, G1212). After DAB development, sections were counterstained with Mayer hematoxylin solution for 10 min. Subsequent gradient dehydration, xylene clearing, and mounting were carried out as follows: 75% ethanol for 5 min, 85% ethanol for 5 min, absolute ethanol I for 5 min, absolute ethanol II for 5 min, n-butanol for 5 min, xylene I for 5 min, followed by mounting with mounting medium. Target protein staining signals and subcellular localization were observed under an Olympus BX53 microscope (Olympus Corp., Japan). No quantitative image analysis was performed in this study; only qualitative evaluation of staining patterns and protein subcellular distribution was conducted.

### 2.23 Bioinformatics Analysis

The ORF information and coding potential prediction data of hsa_circ_0000615 were retrieved from CircBank (http://www.circbank.cn) and CircBase (https://www.circbase.org/). The CircBank platform predicted a cross-junction ORF of 753 nucleotides with maximum coding probability (Coding_Prob = 1), which encodes a 250-amino-acid polypeptide termed ZNF609-250aa. Structural modeling of the polypeptide was not included in the current research system.

The amino acid sequence of human HMGB1 (NP_001300821.1, retrieval date: 2026-08-10) was downloaded from the NCBI database (https://www.ncbi.nlm.nih.gov/), while the polypeptide sequence of ZNF609-250aa encoded by circ_0000615 was retrieved from the CircBank database. The potential binding interfaces between ZNF609-250aa and HMGB1 were predicted by AlphaFold2 (https://alphafold.com/) with default parameters. The generated protein complex structure was imported into PyMOL (Version 2.5.0, Schrödinger, LLC, New York, USA) for visualization, and hydrogen bonds were labeled with yellow dashed lines.

### 2.24 Statistical Analysis

Each experiment was independently repeated three times to ensure reproducibility. Data are presented as mean ± standard error of the mean (SEM). The Shapiro-Wilk test was used to assess the normality of data distribution. Pre-specified two-group comparisons were performed using independent samples *t*-tests for normally distributed data, while the Mann-Whitney U test was adopted for non-normally distributed data. For datasets with three or more experimental groups, one-way ANOVA followed by Tukey’s *post-hoc* multiple-comparison test was used to complete all pairwise comparisons; only biologically relevant pairwise results are annotated in the figures. Two-way repeated-measures ANOVA was applied for longitudinal CCK-8 time-course assays and PDX tumor-growth curve analyses. For the bilateral-tumor PDX model, paired statistical comparisons were performed to account for within-mouse pairing of tumors derived from the same animal. All statistical analyses were conducted with GraphPad Prism 10. All quantitative data are derived from three independent biological experiments. The symbol n represents the number of samples included for analysis. Technical replicates were measured within each biological experiment to ensure measurement reliability. *p* < 0.05 was considered statistically significant (*p < 0.05; **p < 0.01; ***p < 0.001).

## 3 Results

### 3.1 Circ_0615 Is Downregulated in EOC

To identify circRNAs associated with EOC, circRNA-seq was performed on 8 normal ovarian tissues and 10 EOC tissues. Differentially expressed circRNAs were screened with the criteria of |log_2_(FC)| > 0.5 (T vs. N), alongside FDR-adjusted *p* < 0.05 (Fig. 1A), and the top 20 downregulated circRNAs were identified (Fig. 1A). Consistent with the circRNA-Seq results, qRT-PCR showed that circ_0615 expression was significantly lower in 70 EOC tissues than in 80 normal ovarian tissues (Fig. 1B), and was downregulated in EOC cell lines compared with the human normal ovarian epithelial cell line IOSE-80 (Fig. 1C). For survival analysis, patients were stratified into high and low circ_0615 expression groups using the median expression level as the cut-off value. Kaplan-Meier analysis of 70 patients with EOC revealed that elevated circ_0615 expression was associated with improved overall survival (Fig. 1D). These results suggest a potential biological function for circ_0615 in EOC.

Circ_0615 was confirmed as a bona fide circular RNA in other cancers [15], generated from exon 2 of the ZNF609 gene located on human chromosome (chr) 15, and has a length of 874 nt. The back-splice junction (BSJ) site “AGCA” of circ_0615 in EOC cells was amplified using divergent primers, and validated by Sanger sequencing (Fig. 1E), consistent with the circBase database annotation (http://www.circbase.org/). To assess its structural stability, RNase R digestion was employed to demonstrate the resistance of circ_0615 compared to its linear host gene. Divergent primers amplified circ_0615 from cDNA whether or not treated with RNase R, but not from genomic DNA, supporting its circular nature (Fig. 1F). Moreover, actinomycin D treatment for transcription blocking further demonstrated that circ_0615 exhibited greater stability than its linear counterpart ZNF609 mRNA (Fig. 1F). In addition, subcellular localization analysis showed that circ_0615 predominantly localized in the cytoplasm. Both FISH and nuclear-cytoplasmic fractionation followed by qRT-PCR supported this cytoplasmic enrichment, consistent across EOC cell lines (Fig. 1G,H and Fig. S1A).

**Figure 1:**
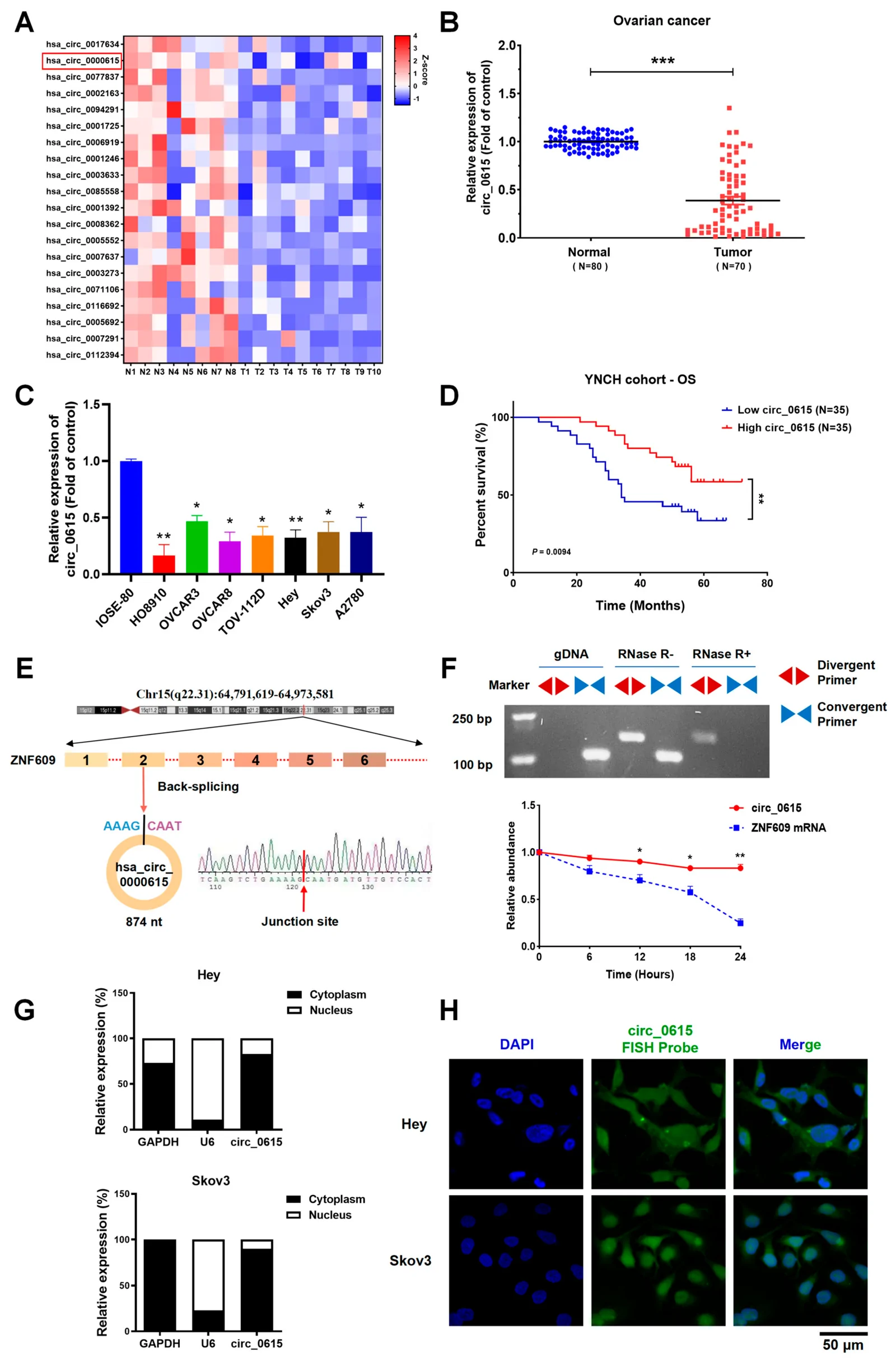
**Characterization of circ_0615 in EOC tissues and cell lines.** (**A**) The hierarchical clustering heatmap shows the 20 most significantly differentially expressed circular RNAs identified in 8 normal ovarian tissues and 10 EOC tissues by circRNA-Seq. DECs were defined as |log_2_FC| > 0.5 and FDR-adjusted *p* < 0.05. (**B**) Detection of circ_0615 expression levels in normal ovarian tissue samples (n = 80) and EOC tissue samples (n = 70) by qRT-PCR. (**C**) qRT-PCR analysis of circ_0615 expression in normal control IOSE-80 cells and EOC cell lines. (**D**) Seventy patients with EOC were stratified into low- and high-circ_0615 expression groups by the median circ_0615 expression level. Kaplan-Meier survival curves were constructed, and the log-rank test was used to compare intergroup overall survival differences. OS, overall survival. (**E**) the diagram shows the genomic location of the ZNF609 gene and splicing patterns of circ_0615, with its splicing junction “AGCA” verified by sanger sequencing. (**F**) Hey cells were treated with or without RNase R, and the amplified products of their cDNA and gDNA were visualized on agarose gel electrophoresis. ZNF609 mRNA and circ_0615 abundance were analyzed by qRT-PCR at the indicated time points treated with act D (2 μg mL^−1^). Statistical analysis was performed using two-way repeated-measures ANOVA. (**G**) Nuclear-cytoplasmic separation assay detecting circ_0615 subcellular localization in hey and Skov3 cell lines. GAPDH and U6 snRNA served as controls for cytosolic and nuclear fractions, respectively. (**H**) FISH was used to detect the subcellular localization of circ_0615 in hey and Skov3 cells. Scale bar = 50 μm. *p < 0.05, **p < 0.01, ***p < 0.001. Three independent experiments were performed.

### 3.2 Circ_0615 Inhibits the Progression of EOC In Vitro and In Vivo

Given the reduced expression of circ_0615 in EOC tissues and cell lines, we hypothesized that circ_0615 may function as a tumor suppressor. Circ_0615 stably overexpressed or knockdown EOC cell lines were generated using a lentiviral system (Fig. 2A,B), and qRT-PCR confirmed efficient modulation of circ_0615 levels without affecting its host gene ZNF609 (Fig. S1B,C). CCK-8 and colony formation assays demonstrated that circ_0615 significantly suppressed cell proliferation and clonogenic capacity, whereas its knockdown produced the opposite effects (Fig. 2C–F). In addition, Transwell and wound-healing assays demonstrated that circ_0615 suppressed the migration of EOC cells, while downregulation of circ_0615 restored the migratory ability of EOC cells (Fig. 2G,H and Fig. S2A,B). Moreover, flow cytometric analysis showed that circ_0615 promoted cell apoptosis, whereas silencing circ_0615 led to a significant reduction in apoptotic rates (Fig. S2C,D).

To confirm the tumor-suppressive effect of circ_0615 *in vivo*, a subcutaneous xenograft model was established using BALB/c nude mice. Mice were inoculated with Hey cells stably overexpressing circ_0615 or circ_NC. The results demonstrated that circ_0615 significantly inhibited tumor growth (Fig. 2I,J), accompanied by reduced tumor weight (Fig. 2K) and elevated circ_0615 expression levels (Fig. 2L). H&E staining and IHC revealed that circ_0615 restrained proliferative activity within EOC xenograft tissues (Fig. S2E). Collectively, these findings suggest that circ_0615 may function as a tumor suppressor in EOC by inhibiting proliferation and migration while promoting apoptosis.

**Figure 2:**
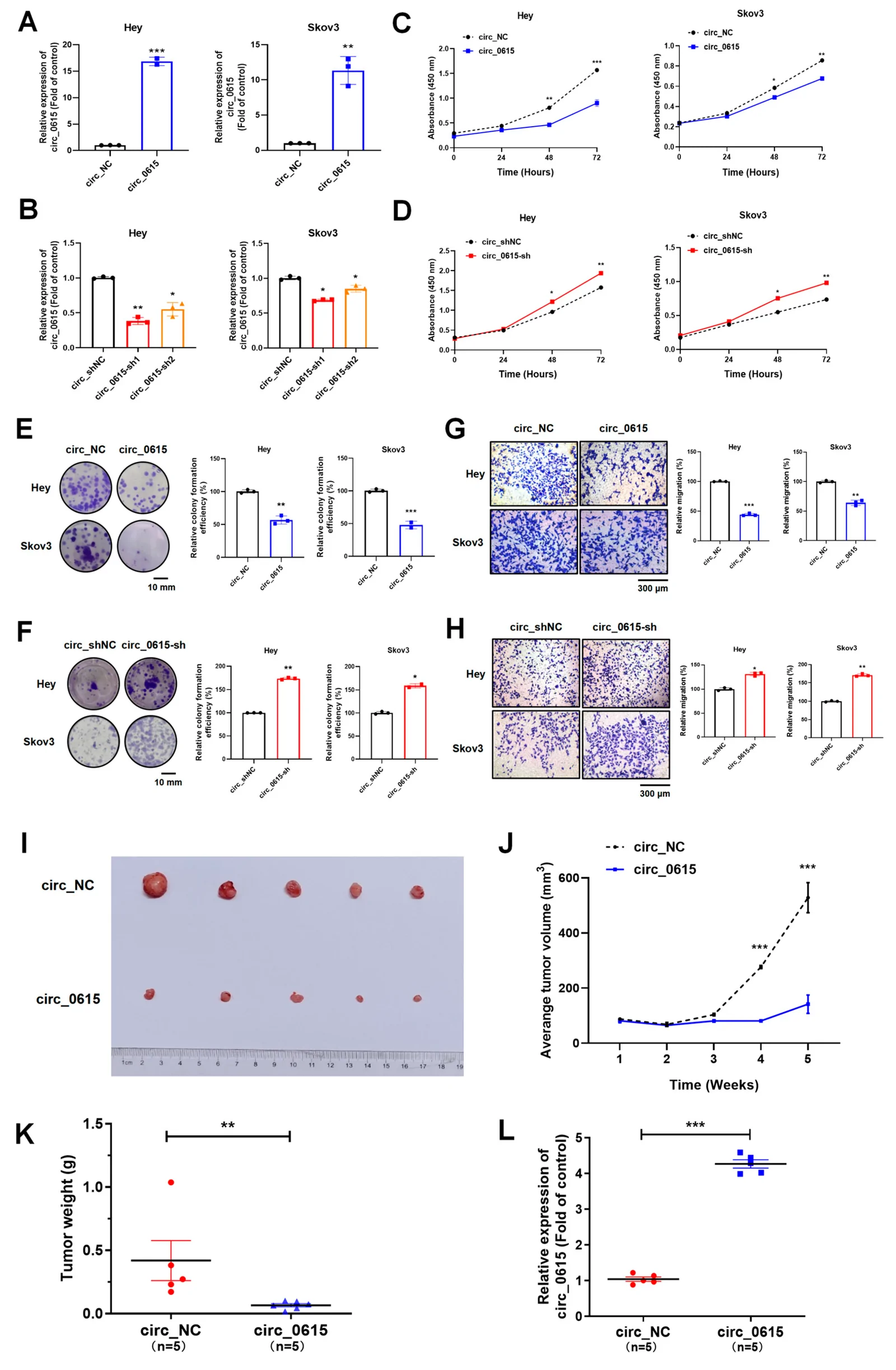
**Circ_0615 inhibits EOC proliferation and migration *in vitro* and suppresses tumor growth *in vivo*.** (**A**) qRT-PCR detection of circ_0615 expression in circ_0615-overexpressing EOC cells and circ_NC controls. (**B**) qRT-PCR detection of circ_0615 expression in circ_0615-sh-transfected EOC cells and circ_shNC controls. (**C**) CCK-8 assay was performed to assess proliferation in circ_0615-overexpression EOC cells and circ_NC control. Statistical analysis was performed using two-way repeated-measures ANOVA. (**D**) CCK-8 assay was performed to assess proliferation in circ_0615-silenced EOC cells and circ_shNC control. Statistical analysis was performed using two-way repeated-measures ANOVA. (**E**) Colony-formation assay was performed to assess the proliferative capacity of EOC cells with circ_0615 overexpression versus circ_NC control. Scale bar = 10 mm. (**F**) Colony-formation assay was performed to assess the proliferative capacity of EOC cells with circ_0615 knockdown versus circ_shNC control. Scale bar = 10 mm. (**G**) Transwell assay for migration capacity of circ_0615-overexpressing EOC cells and circ_NC controls. Scale bar = 300 μm. (**H**) Transwell assay for migration capacity of circ_0615-silenced EOC cells and circ_shNC controls. Scale bar = 300 μm. (**I**) Representative images of subcutaneous xenograft tumors derived from circ_0615-overexpressing EOC cells and circ_NC controls in nude mice (n = 5). (**J**) Weekly measurement of subcutaneous tumor volumes for five consecutive weeks in xenograf-bearing nude mice. Statistical analysis was performed using two-way repeated-measures ANOVA. (**K**) Final weight of xenograft tumors from circ_0615-overexpressing group and circ_NC group (n = 5). (**L**) qRT-PCR analysis of circ_0615 expression in harvested xenograft tumor tissues (n = 5). *p < 0.05, **p < 0.01, ***p < 0.001. Three independent experiments were performed.

### 3.3 Circ_0615 Encodes the Polypeptide ZNF609-250aa

Given that circRNAs can function either by interacting with RNA-binding proteins or by encoding peptides, we assessed the potential coding capacity of circ_0615 in EOC. Bioinformatic analysis of public circRNA databases (CircBank and CircBase) predicted a 753-nt open reading frame (ORF) spanning the back-splicing junction of circ_0615, which theoretically encodes a 250aa polypeptide (ZNF609-250aa) (Fig. S3A). To validate the coding capacity of ZNF609-250aa, we constructed a circ_0615 expression vector containing a Flag tag (circ_0615-Flag), a linear expression vector encoding the 250-aa polypeptide from the 753-nt ORF of circ_0615 with three Flag tags (ZNF609-250aa-Flag), and a mutant circ_0615 vector (circ_0615-mut), in which the native start codon ATG of the circ_0615 ORF was mutated to ACG to abolish translation initiation (Fig. 3A–C). Stable overexpression of circ_0615-Flag and circ_0615-mut both increased circ_0615 abundance without altering the expression of its host gene ZNF609 (Fig. S3B,C). A ~35 kDa band was detected in circ_0615-Flag EOC cells, and a ~38 kDa band was detected in ZNF609-250aa-Flag EOC cells, both corresponding to ZNF609-250aa, whereas no band was observed in circ_0615-mut EOC cells (Fig. 3A–C). The canonical linear ZNF609 protein is a 1411-aa product with partial sequence overlap with the circ_0000615-translated polypeptide. We therefore subjected the ~34 kDa protein from circ_0615-overexpressing EOC cells to LC-MS/MS, and peptides corresponding to the circ_0615-encoded polypeptide were detected (Fig. 3D, Table S3). In addition, ZNF609-250aa expression positively correlated with circ_0615 levels (Fig. 3E). Importantly, compared with normal tissues, ZNF609-250aa expression was reduced in EOC tissues, and a similar trend was observed in EOC cell lines (Fig. S3D,E), consistent with the expression of circ_0615. Together, these data suggest that circ_0615 encodes the ZNF609-250aa polypeptide in EOC and indicate that this polypeptide may function as tumor-suppressor.

To further elucidate the mechanism by which circ_0615-encoded ZNF609-250aa functions in EOC, an RNA pull-down assay was performed to identify proteins associated with circ_0615 that may contribute to its translation. Biotin-labeled probes revealed a specific ~35 kDa protein band interacting with circ_0615, and subsequent mass spectrometry identified seven associated proteins, including eIF2α (Fig. 3F and Fig. S3F). Moreover, RIP-qRT-PCR and western blot analyses confirmed the interaction between circ_0615 and eIF2α in EOC cells (Fig. 3G and Fig. S3G). Notably, eIF2α knockdown significantly reduced ZNF609-250aa levels in circ_0615-Flag EOC cells. Meanwhile, circ_0615 expression remained unchanged upon eIF2α silencing, indicating that eIF2α associates with circ_0615 and is required for efficient ZNF609-250aa expression (Fig. 3H and Fig. S3H).

**Figure 3:**
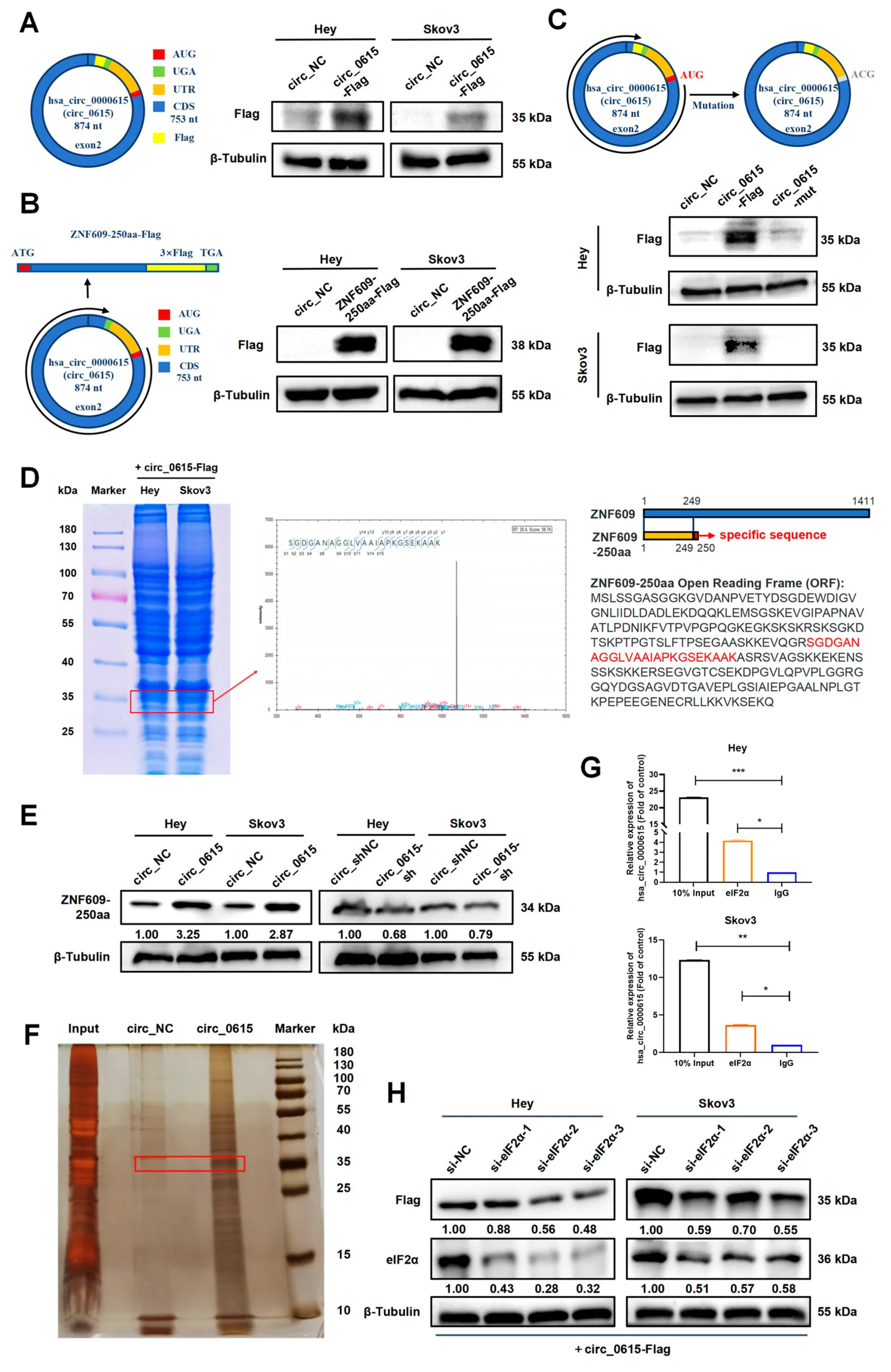
**Circ_0615 binds to eIF2α and encodes the ZNF609-250aa polypeptide.** (**A**) Schematic representation of the circ_0615-Flag expression vector, with western blot detection of Flag-tagged protein post-transfection. (**B**) Schematic illustration of the linear ZNF609-250aa-Flag expression vector, accompanied by western blot verification of Flag-tagged protein expression. (**C**) Schematic diagram of circ_0615-mut vector carrying a mutated ORF start codon, plus western blot analysis for Flag-tagged protein upon transfection. (**D**) SDS-PAGE gels from circ_0615-Flag hey and Skov3 cells were stained with coomassie brilliant blue. The red box indicates the band corresponding to the flag tag, wherein mass spectrometry analysis detected a polypeptide sequence (red) within ZNF609-250aa. (**E**) Western blot analysis of ZNF609-250aa levels in circ_0615 or circ_0615-sh EOC cells. (**F**) Silver-stained SDS-PAGE gel displays circ_0615-binding proteins in the RNA pulldown. The red box indicates the specific protein band (~35 kDa) identified by comparison with the circ_NC probe, which was subsequently subjected to LC-MS/MS analysis. (**G**) The fold enrichment of circ_0615 binding to eIF2α relative to the IgG control was detected by RIP. (**H**) Western blot analysis of ZNF609-250aa levels following eIF2α knockdown in hey and Skov3 cells. *p < 0.05, **p < 0.01, ***p < 0.001. Abbreviation: AUG, start codon; UGA, stop codon; ATG, start codon; TGA, stop codon; UTR, untranslated region; CDS, coding sequence; flag, FLAG tag.

### 3.4 Circ_0615 Inhibits the Progression of EOC In Vitro and In Vivo by Encoding the ZNF609-250aa Polypeptide

Given that circ_0615 and its encoded ZNF609-250aa have been implicated in the pathogenesis of multiple diseases, we further investigated the function of ZNF609-250aa in EOC cell lines. Functional assays, including CCK-8 and colony formation assays, demonstrated that overexpression of circ_0615 or ZNF609-250aa significantly suppressed cell proliferation, whereas circ_0615-mut showed no appreciable effect (Fig. 4A–D). Moreover, Transwell assays showed that both circ_0615 and ZNF609-250aa inhibited the migratory capacity of EOC cells, whereas overexpression of circ_0615-mut exhibited no suppressive effect (Fig. 4E,F). To confirm that the oncogenic phenotypes triggered by circ_0615 silencing arise from reduced ZNF609-250aa production, we carried out rescue experiments in Hey and SKOV3 cells. According to CCK-8 and colony formation assays, overexpression of ZNF609-250aa in circ_0615-knockdown cells not only reversed the pro-proliferative phenotype induced by circ_0615 depletion, but led to further suppressed cell proliferation compared with circ_NC group (Fig. 4G–I). Collectively, these rescue data indicate that the phenotypes caused by circ_0615 depletion are predominantly mediated by loss of the ZNF609-250aa polypeptide.

**Figure 4:**
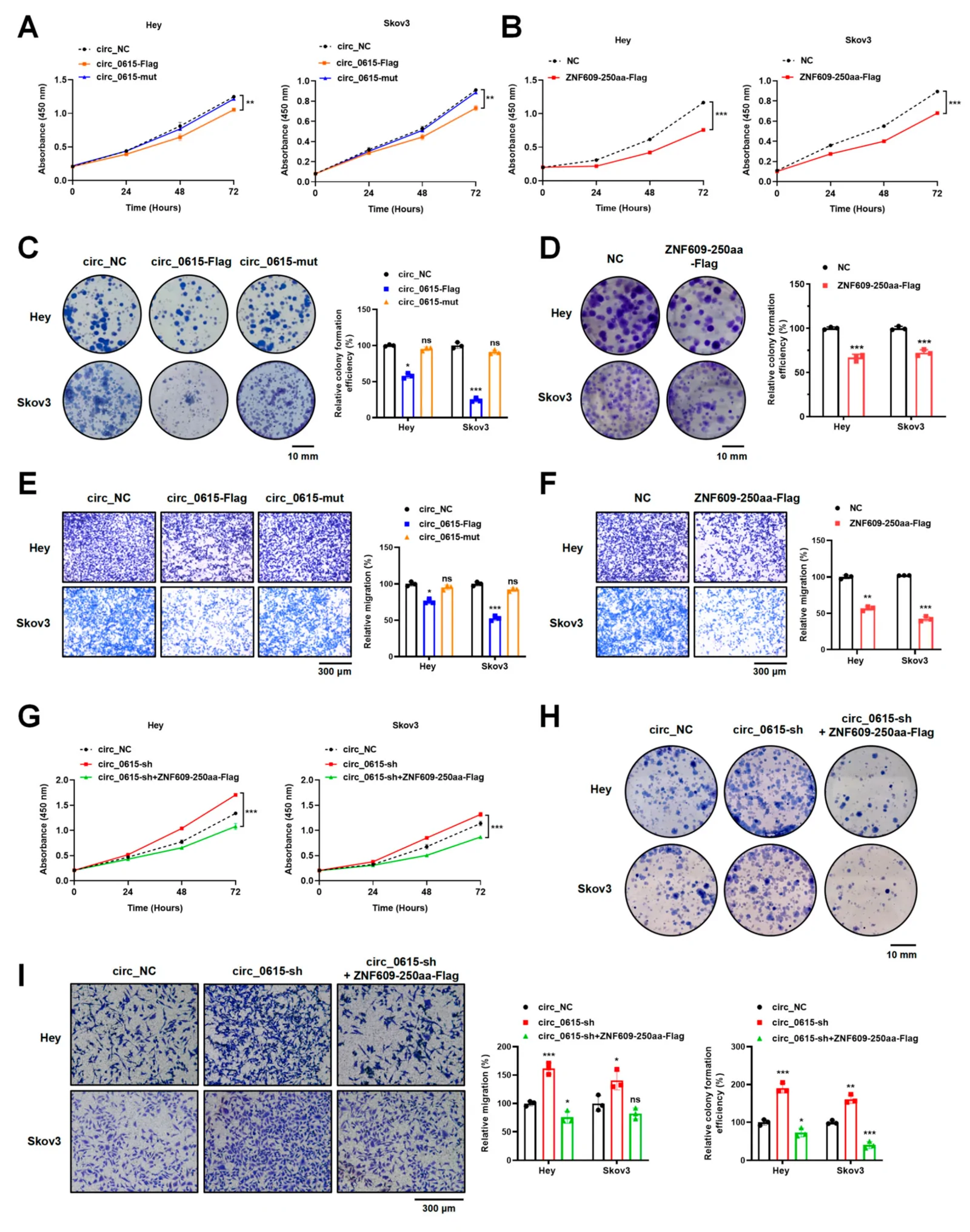
ZNF609-250aa polypeptide suppresses hey and Skov3 cell proliferation *in vitro*. (**A**) CCK-8 assay for viability of EOC cells transfected with circ_NC, circ_0615-Flag or circ_0615-mut. Statistical analysis was performed using two-way repeated-measures ANOVA. Significance is only indicated for the circ_0615-Flag versus circ_0615-mut comparison. (**B**) CCK-8 assay for viability of EOC cells transfected with empty vector NC and ZNF609-250aa-Flag. Statistical analysis was performed using two-way repeated-measures ANOVA. (**C**) Colony-formation assay for proliferative capacity of EOC cells stably transfected with circ_NC, circ_0615-Flag or circ_0615-mut. Scale bar = 10 mm. (**D**) Colony-formation assay for proliferative capacity of EOC cells stably transfected with empty-vector NC and ZNF609-250aa-Flag. Scale bar = 10 mm. (**E**) Transwell migration assay for migratory ability of EOC cells stably transfected with circ_NC, circ_0615-Flag or circ_0615-mut. Scale bar = 300 μm. (**F**) Transwell migration assay for migratory ability of EOC cells stably transfected with empty-vector NC and ZNF609-250aa-Flag. Scale bar = 300 μm. (**G**) CCK-8 assay for proliferation of circ_NC, circ_0615-sh and circ_0615-sh+ZNF609-250aa-Flag EOC cells. Statistical analysis was performed using two-way repeated-measures ANOVA. Significance is only indicated for the circ_0615-sh versus circ_0615-sh+ZNF609-250aa-Flag comparison. (**H**) Colony-formation assay for proliferative capacity of circ_NC, circ_0615-sh and circ_0615-sh+ZNF609-250aa-Flag EOC cells. Scale bar = 10 mm. (**I**) Transwell migration assay for migratory ability of circ_NC, circ_0615-sh and circ_0615-sh+ZNF609-250aa-Flag EOC cells. Scale bar = 300 μm. *p < 0.05, **p < 0.01, ***p < 0.001, ns, *p* > 0.05. Three independent experiments were performed.

To further validate the tumor-suppressive function of circ_0615 encoded ZNF609-250aa in EOC, we established PDX models (Fig. S4A). Recombinant AAV2/8 vectors encoding ZNF609-250aa or a negative control (NC) were constructed. AAV2/8-ZNF609-250aa and AAV2/8-NC virions were intraperitoneally injected into PDX mouse models for seven consecutive days. AAV-mediated GFP signaling was monitored by IVIS imaging to assess tumor progression. Compared with the control group, AAV2/8-ZNF609-250aa treatment significantly suppressed tumor growth and reduced tumor volume and weight (Fig. 5A–D). Frozen-section GFP imaging, where GFP serves as an AAV-transduction reporter, confirmed effective intraperitoneal transduction of AAV vectors in PDX tumor tissue (Fig. 5E). Together, these findings suggest that circ_0615 encodes the ZNF609-250aa polypeptide, which potently suppresses tumor cell proliferation and migration *in vitro* and inhibits tumor progression *in vivo*, supporting its value as a candidate target worthy of further preclinical investigation in EOC.

**Figure 5:**
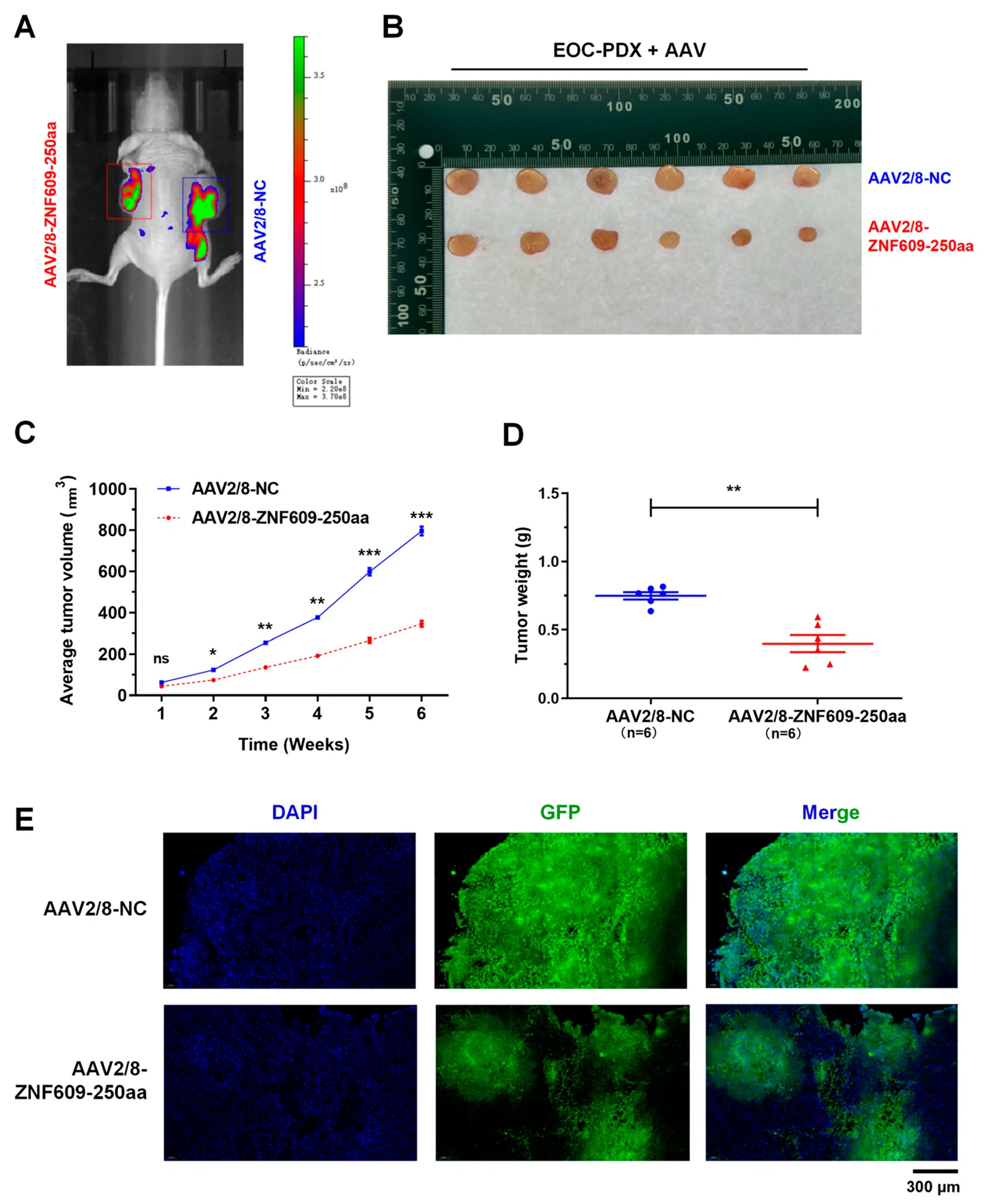
ZNF609-250aa inhibits tumor growth in the EOC-PDX model. (**A**) Representative image of AAV-mediated GFP expression signals in tumors detected using the IVIS 1501 imaging system. (**B**) Tumor images of each group 42 days after intraperitoneal injection of AAV2/8-NC or AAV2/8-ZNF609-250aa (n = 6). (**C**) Subcutaneous tumor volumes were measured weekly for six consecutive weeks (n = 6). (**D**) Final tumor weights were recorded after tumor resection (n = 6). (**E**) Representative GFP fluorescence images of frozen sections derived from AAV-NC and AAV-ZNF609-250aa treated EOC PDX tumors, verifying successful intraperitoneal AAV transduction. Scale bar = 300 μm. *p < 0.05, **p < 0.01, ***p < 0.001, ns, *p* > 0.05. Three independent experiments were performed.

### 3.5 ZNF609-250aa Interacts with HMGB1 and Increases Its Levels in EOC Cells

Co-IP combined with mass spectrometry was performed to identify potential ZNF609-250aa–interacting proteins and to elucidate the molecular mechanisms underlying its function in EOC. Protein A/G magnetic beads conjugated with Flag antibodies were used to enrich interacting proteins from ZNF609-250aa-Flag EOC cells, followed by SDS–PAGE and silver staining. A distinct ~25-kDa band was observed, and mass spectrometry identified HMGB1 as a potential binding partner of ZNF609-250aa (Fig. 6A). Moreover, Co-IP assays demonstrated that immunoprecipitates pulled down by either Flag or HMGB1 antibodies contained both proteins, confirming their interaction (Fig. 6B). Consistently, AlphaFold structural modeling predicted potential interaction interfaces between ZNF609-250aa and HMGB1 (Fig. 6C). To further validate this interaction in EOC cells, IF analysis showed that ZNF609-250aa and HMGB1 co-localized in the cytoplasm (Fig. 6D), and PLA revealed distinct fluorescent puncta, indicating their spatial proximity and physical association (Fig. 6E). Collectively, these results suggest that ZNF609-250aa physically interacts with HMGB1 in EOC cells, which may contribute to its anti-cancer properties.

**Figure 6:**
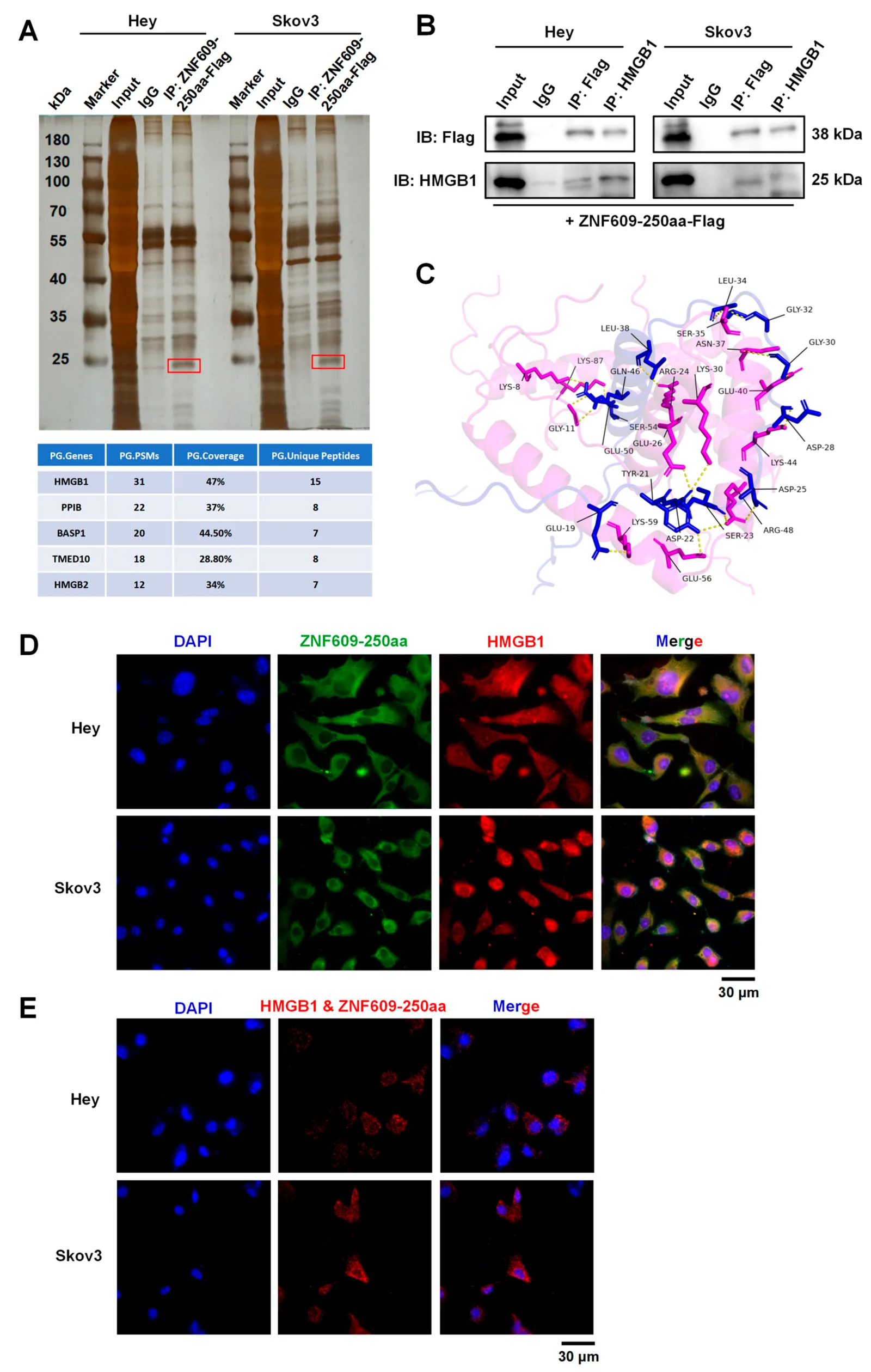
**ZNF609-250aa interacts with HMGB1 in EOC cells.** (**A**) The silver-stained SDS-PAGE gel image shows ZNF609-250aaFlag-binding proteins in Co-IP. The red box indicates the specific protein band (~25 kDa) identified by comparison with the IgG, which was subsequently subjected to LC-MS/MS analysis. Five potential binding proteins identified by LC-MS/MS analysis are presented in the table. (**B**) Reciprocal Co-IP assay verifying the interaction between ZNF609-250aa and HMGB1 in hey and Skov3 cells. (**C**) The binding site between ZNF609-250aa and the HMGB1 amino acid sequence was predicted using AlphaFold, with its 3D model displayed in pymol. Yellow dashed lines indicate hydrogen bonds. (**D**) Subcellular co-localization of ZNF609-250aa and HMGB1 detected by IF assays in hey and Skov3 cells. Scale bar = 30 μm. (**E**) Close spatial proximity between ZNF609-250aa and HMGB1 detected by PLA assays in hey and Skov3 cells. Scale bar = 30 μm. Three independent experiments were performed.

Subsequently, we examined the function of the interaction between ZNF609-250aa and HMGB1 by assessing HMGB1 expression changes in EOC cells. Overexpression of ZNF609-250aa significantly increased HMGB1 protein levels but not mRNA levels (Fig. S4B). Furthermore, HMGB1 levels were increased in circ_0615 overexpressing EOC cells, whereas they were downregulated in the circ_0615-sh cell line. However, transient expression of ZNF609-250aa restored HMGB1 levels in circ_0615-sh EOC cells, while HMGB1 mRNA levels remained unchanged (Fig. S4C–E). In addition, IHC staining of PDX tumor tissues showed stronger HMGB1 staining and weaker Ki-67 staining in the AAV2/8-ZNF609-250aa group (Fig. S4F). Collectively, these findings suggest that ZNF609-250aa specifically enhances HMGB1 protein abundance both *in vitro* and *in vivo*.

### 3.6 ZNF609-250aa Affects the Expression Level and Nuclear-Cytoplasmic Distribution of HMGB1 in EOC Cells

Given that ZNF609-250aa interacts with HMGB1 and upregulates HMGB1 protein levels rather than mRNA levels, we hypothesized that ZNF609-250aa may stabilize HMGB1 by modulating ubiquitin-proteasome-mediated degradation or autophagy. To confirm this hypothesis, we employed CHX to block *de novo* protein synthesis, MG-132 to inhibit proteasomal degradation, and chloroquine (CQ) to suppress autophagy-lysosomal degradation. Mechanistically, CHX chase assays suggest that ZNF609-250aa improves HMGB1 protein stability (Fig. 7A). In control cells, MG132 treatment upregulated HMGB1 levels; however, MG132 failed to further elevate HMGB1 abundance in ZNF609-250aa-overexpressing cells. Meanwhile, CQ induced only minor changes in HMGB1 expression in both groups (Fig. 7B). These data support that HMGB1 degradation mainly relies on the proteasome pathway. Consistently, Co-IP analysis, performed under denaturing conditions for ubiquitination detection, revealed reduced ubiquitination of HMGB1 in the presence of ZNF609-250aa (Fig. 7C). As molecular chaperones have been shown to regulate HMGB1 subcellular localization through ubiquitination [43], we further determined whether ZNF609-250aa affects HMGB1 distribution within the nucleus and cytoplasm, which is critical for its biological functions. Nuclear-cytoplasmic fractionation and IF assays revealed increased nuclear HMGB1 signals upon ZNF609-250aa expression (Fig. 7D,E). This alteration could stem from elevated total HMGB1 abundance and/or changes in subcellular partitioning. Collectively, these findings support that ZNF609-250aa stabilizes HMGB1 by restraining ubiquitin–proteasome degradation and is associated with increased nuclear HMGB1 levels, revealing functional crosstalk between ZNF609-250aa and HMGB1.

**Figure 7:**
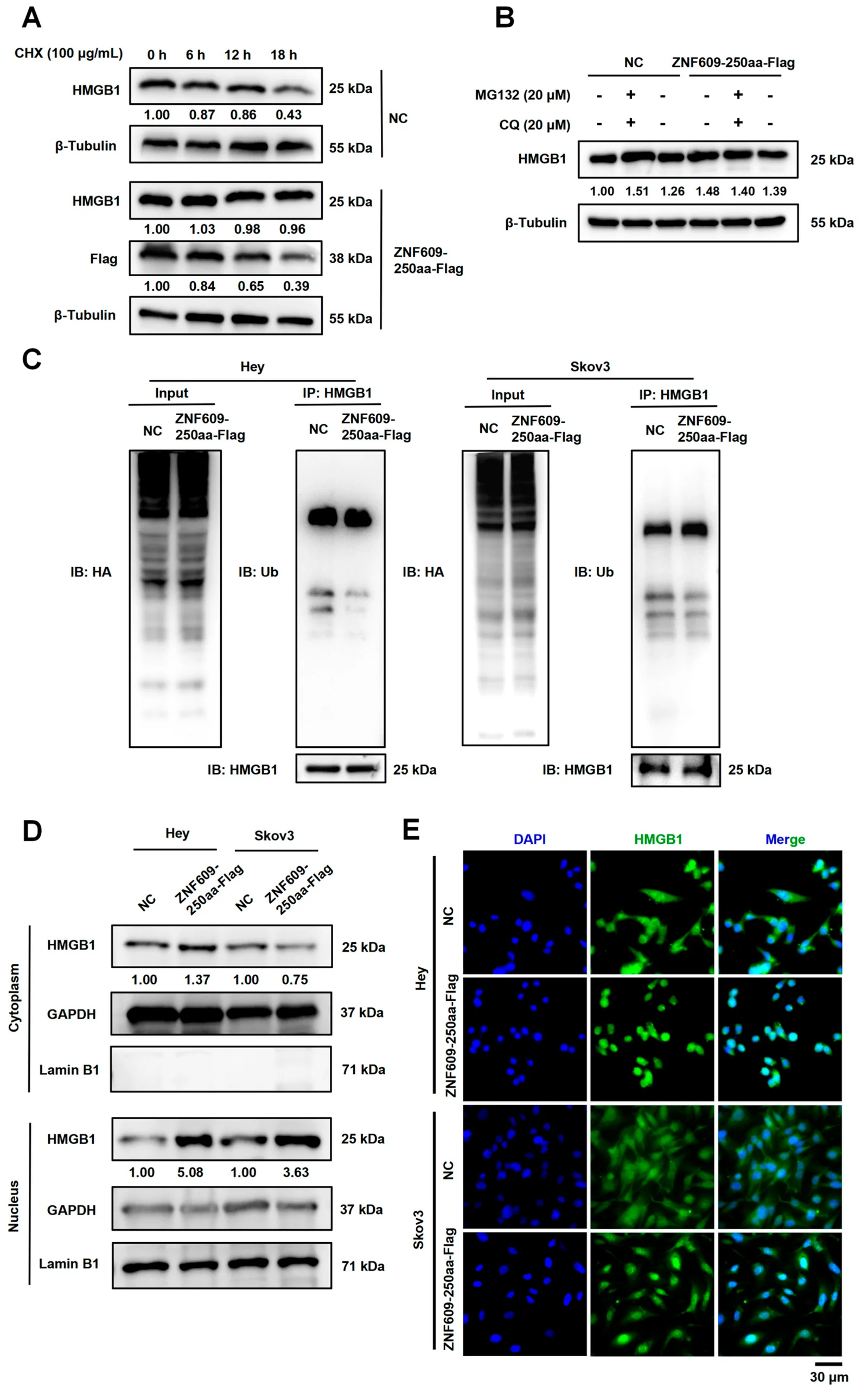
**ZNF609-250aa polypeptide stabilizes the expression of HMGB1 in EOC cell lines.** (**A**) ZNF609-250aa-Flag hey cells and control group were treated with 100 μg mL^−1^ of CHX, and total proteins were extracted at 0 h, 6 h, 12 h, and 18 h, respectively, for western blot analysis. (**B**) Western blot analysis of HMGB1 protein levels in NC and ZNF609-250aa-Flag hey cells treated with vehicle, MG132, or CQ (20 μM for 8 h). (**C**) Equal amounts of HA-Ub plasmid were transfected into hey and Skov3 cell lines transfected with ZNF609-250aa-Flag or controls, and the ubiquitination level of HMGB1 was analyzed by Co-IP with western blot after MG-132 treatment. (**D**) Cytoplasmic and nuclear proteins were isolated by subcellular fractionation, and western blot analysis was performed to assess HMGB1 expression levels within EOC cells. (**E**) Subcellular co-localization of HMGB1 detected by IF assays in ZNF609-250aa-Flag EOC cells or the control group. Scale bar = 30 μm. Three independent experiments were performed.

### 3.7 ZNF609-250aa Facilitates Nuclear HMGB1 to Regulate ALDH1A1 Transcription in EOC Cells

To investigate the nuclear function of HMGB1 in EOC cells following ZNF609-250aa dysregulation, we performed ChIP-seq and mRNA-seq analyses in ZNF609-250aa-Flag EOC cells (Fig. 8A,B). Integrated analysis identified ALDH1A1, a well-established cancer stem cell marker, as the most significantly enriched downstream gene regulated by ZNF609-250aa. ChIP-seq results revealed increased HMGB1 occupancy at the ALDH1A1 promoter region (Fig. 8A), which was further validated by ChIP-qRT-PCR showing enhanced HMGB1 enrichment upon ZNF609-250aa overexpression (Fig. 8B). Moreover, mRNA-seq screening of differentially expressed genes following ZNF609-250aa upregulation, together with subsequent validation at both the mRNA and protein levels, confirmed the consistent downregulation of ALDH1A1 (Fig. 8C–E). To determine whether ALDH1A1 is directly regulated by HMGB1, HMGB1 was silenced in ZNF609-250aa-Flag EOC cell lines, which restored ALDH1A1 expression at both mRNA and protein levels (Fig. 8F,G and Fig. S4G). Collectively, these findings suggest that ZNF609-250aa promotes the nuclear accumulation of HMGB1, enabling HMGB1 binding to the ALDH1A1 promoter and resulting in transcriptional repression of ALDH1A1 in EOC cells. This indicates one potential downstream signalling branch linking the ZNF609-250aa/HMGB1 cascade to altered tumor cell behaviours.

**Figure 8:**
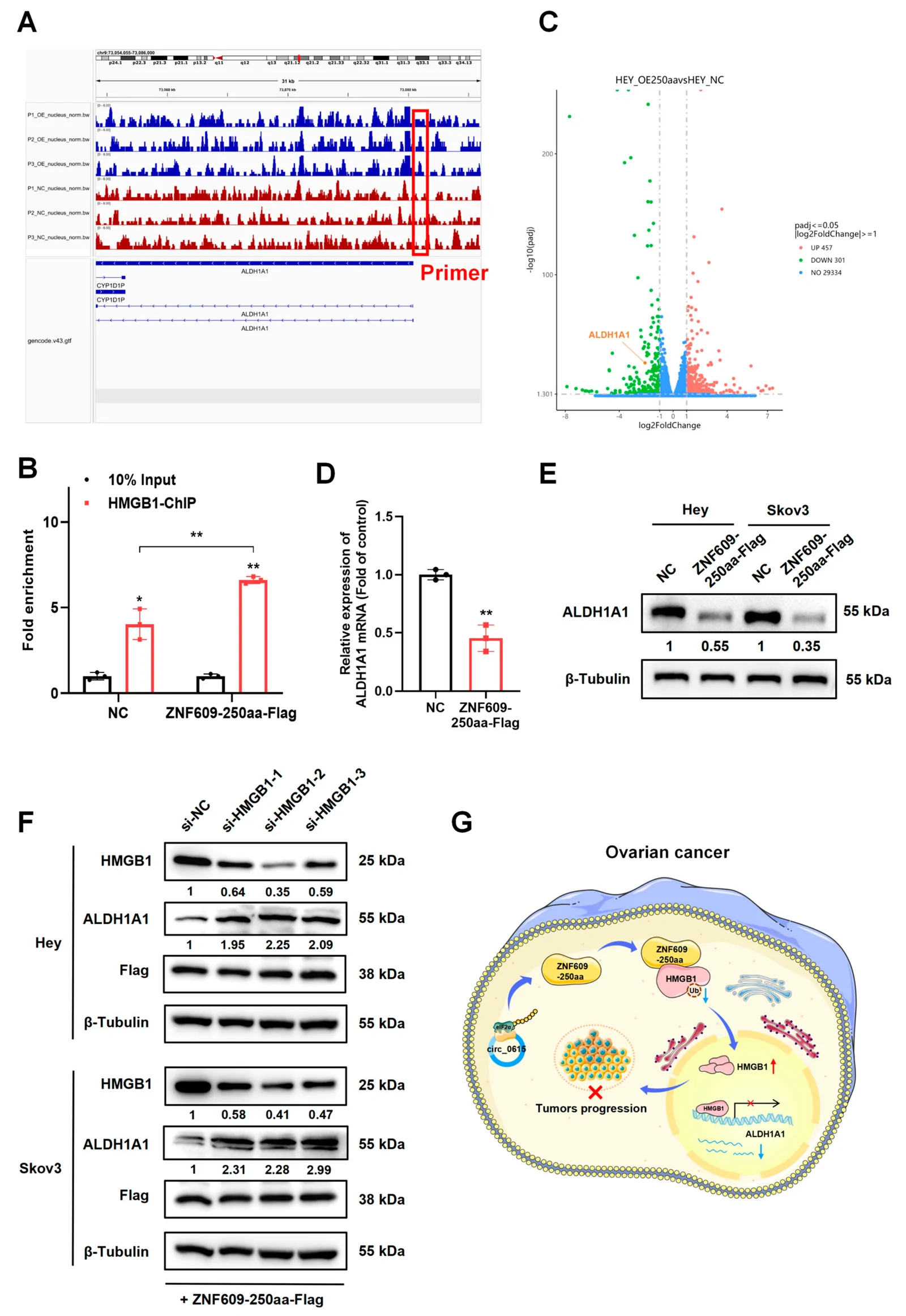
**ZNF609-250aa regulates ALDH1A1 levels through HMGB1.** (**A**) IGV track plot illustrating ChIP-seq enrichment signals of HMGB1 within the promoter region of ALDH1A1. Blue peaks represent control cells, and red peaks represent ZNF609-250aa-Flag hey cells. (**B**) ChIP-qPCR analysis with primers specific for the HMGB1-ALDH1A1 binding region was employed, and the enrichment level of HMGB1 on ALDH1A1 DNA was assessed in ZNF609-250aa-Flag EOC cells and control cells. (**C**) The volcano plot of mRNA-seq shows differentially expressed genes between the control and the ZNF609-250aa-Flag hey cells. (**D**) qRT-PCR analysis of ALDH1A1 mRNA levels in ZNF609-250aa-Flag hey cells and control group. (**E**) Western blot analysis of ALDH1A1 protein levels in ZNF609-250aa-Flag EOC cell lines and control cells. (**F**) Western blot analysis of HMGB1 and ALDH1A1 RNA and protein levels following HMGB1 knockdown in ZNF609-250aa-Flag EOC cell lines. (**G**) The graphic illustrates that ZNF609-250aa inhibits EOC progression via binding to HMGB1 and reducing its ubiquitination levels, and is associated with increased nuclear HMGB1 levels. *p < 0.05, **p < 0.01. Three independent experiments were performed.

## 4 Discussion

Traditionally, circRNAs have been recognized to function primarily as miRNA sponges or through interactions with RNA-binding proteins to modulate gene expression [21]. However, not all circRNAs possess abundant miRNA-binding sites, suggesting the existence of additional regulatory mechanisms. Increasing evidence indicates that certain circRNAs harbor ORFs, IRES elements, or m^6^A modifications, which enable their translation into functional peptides that exert regulatory functions in multiple cancers [44,45,46]. However, the translational capacity of circRNAs and the functions of their encoded peptides remain poorly understood in OC. Previous studies have shown that circ_0615 can exert either oncogenic or tumor-suppressive effects in different cancers, while its encoded polypeptide, ZNF609-250aa, has been implicated in kidney injury and muscle proliferation [21,22]. In the present study, circ_0615 was found to be downregulated in EOC and exhibited a stable circular structure that enables the encoding of the cytoplasmic polypeptide ZNF609-250aa. These findings suggest that circ_0615 can stably encode ZNF609-250aa and has distinct biological functions under different pathological conditions. Additionally, previous studies have demonstrated that the translation of ZNF609 in muscle cells correlates with eIF2α activity [21]. Consistently, we observed that circ_0615 interacts with eIF2α, and knockdown of eIF2α significantly reduced the expression of circ_0615-encoded ZNF609-250aa in EOC cells, suggesting a potential conserved role of eIF2α in supporting circRNA-encoded peptide production in eukaryotes. Notably, disruption of the peptide-coding capacity of circ_0615 suggests that circ_0615 suppresses *in vitro* EOC progression predominantly through encoding ZNF609-250aa, although potential RNA-mediated functions cannot be fully excluded. Furthermore, AAV-mediated overexpression of ZNF609-250aa effectively suppressed tumor growth in PDX mouse models. These preclinical observations support further investigation into circRNA-encoded peptides as candidate molecular targets for EOC intervention, though additional studies are essential to evaluate their translational prospects.

HMGB1 primarily functions as a nucleoprotein. Recent research has revealed that nuclear export of HMGB1 interferes with cytoplasmic autophagy and apoptosis, and HMGB1 secreted into the extracellular space can modulate antitumor immune responses under stress conditions [29,47]. Ubiquitin–proteasome–mediated degradation is one of the key mechanisms regulating HMGB1 subcellular localization and enabling these diverse functions. Notably, ubiquitin-specific proteasome subunits 13 (USP13) and USP22 have been identified as molecular chaperones of HMGB1. By preventing ubiquitin-mediated degradation, they stabilize HMGB1 and promote its nuclear–cytoplasmic translocation and secretion [48,49]. In contrast, the E3 ubiquitin ligases RNF125 and RNF186 facilitate ubiquitin-dependent degradation of cytoplasmic HMGB1, thereby suppressing autophagy [43,50]. Notably, our study found that ZNF609-250aa interacts with HMGB1, reduces its ubiquitination, and thereby enhances HMGB1 protein stability and abundance. However, whether ZNF609-250aa directly interferes with E3 ligase recruitment or alters HMGB1 conformation to prevent ubiquitination remains to be elucidated. Additionally, we observed that ZNF609-250aa significantly promotes nuclear accumulation of HMGB1, rather than facilitating its secretion, thereby enabling HMGB1 to function predominantly as a nuclear protein. Potential mechanisms include enhanced interaction of HMGB1 with nuclear import proteins or exposure of its nuclear localization signal upon reduced ubiquitination [51]. These hypotheses warrant further validation using NLS-deficient mutants and proteomic analyses.

As a chromatin-associated protein, nuclear HMGB1 may influence transcriptional repression either by competitively binding to DNA promoter regions, thereby preventing transcription factor recruitment, or by stabilizing inactive chromatin conformations, such as closed nucleosome states, which indirectly inhibit gene expression [52,53]. Previous studies have also demonstrated that HMGB1 can suppress the activity of the IKB-α promoter [54]. Consistent with these functions, our results showed that HMGB1 binds to the ALDH1A1 promoter and suppresses its transcription, potentially explaining how HMGB1 impairs cancer stemness and malignant progression in EOC. Research indicates that ALDH1A1 may contribute to acquired cisplatin resistance in OC by upregulating NEK-2 (Never in Mitosis Gene A-related Kinase 2) [55]. Furthermore, ALDH1A1 inhibitors can effectively suppress ovarian carcinogenesis by mediating nuclear factor erythroid 2-related factor 2 (NRF2) silencing, whereas nuclear HMGB1 inhibits NRF2 activation and accumulation, suggesting a potential association between ALDH1A1 and HMGB1 within the nucleus [56,57,58]. Although studies have proposed that HMGB1 may act as a transcription factor chaperone and enhance transcription [59], whether HMGB1 inhibits ALDH1A1 by competing with transcriptional activators remains to be determined. Importantly, ALDH1A1 serves as only one plausible downstream target of the ZNF609-250aa/HMGB1 axis in this study, and further phenotypic rescue experiments are still required to clarify its independent functional contribution to EOC malignant progression, as multiple downstream effectors may collaboratively mediate the full spectrum of tumor phenotypes.

In summary, our study provides novel evidence in EOC that circ_0615 encodes ZNF609-250aa in EOC and exerts tumor-suppressive effects *in vivo* and *in vitro* via this peptide. Mechanistically, circ_0615 binds eIF2α, which is required for efficient ZNF609-250aa production, and the resulting ZNF609-250aa interacts with HMGB1. This interaction enhances HMGB1 stability and nuclear accumulation by reducing ubiquitination, which is associated with repression of ALDH1A1 transcriptional activity in the nucleus, and may contribute to EOC-inhibitory phenotypes, ultimately inhibiting EOC progression. These obtained results extend the functional repertoire of circRNAs beyond ceRNA mechanisms, highlighting their potential as a novel source of functional peptides and promising pre-clinical therapeutic targets for EOC that require further validation. One limitation of this retrospective study is that perfect age-and menopause-status-matching could not be achieved for the normal ovarian control group owing to the constraints of available clinical specimens.

## Data Availability

The circRNA expression of tissues has been deposited on the National Genomics Data Center and is available under the accession number OMIX019694. Additional data are available from the corresponding author on reasonable request.

## Supplementary Materials

Supporting Information document, including Figs. S1–S4 and Tables S1–S3. The supplementary material is available online at https://www.techscience.com/doi/10.32604/or.2026.087931/s1.
