# Supplementary material for: Hsa_Circ_0000615-Encoded 250-Amino Acid Polypeptide Suppresses Epithelial Ovarian Cancer Progression via Promoting HMGB1 Nuclear Accumulation
Source: Oncol Res. 2026 Oct 28;34(11):22. doi: 10.32604/or.2026.087931 (PMC13732393; doi:10.32604/or.2026.087931)
Supplement: Supplementary file 1 [file OncolRes-34-87931-s001.zip › TSP_OR_87931_Supplementary_Files.docx]

Supporting information

**Hsa_circ_0000615-encoded 250-amino acid polypeptide suppresses epithelial ovarian cancer progression via promoting HMGB1 nuclear accumulation**

*ZiYi Guo^1#^, SiYun Deng^1#^, Jiao Meng^3#^, XiaoFei Zhang^4^, YueYou Wang^1^, JiaMi Huang^5^, TongLu Li^6^, Min Liu^1^, HengHui Yang^2^, FangZhou Chen^2^, Yuan Wang^1^, Wei Meng^1,7*^, ShaoJia Wang^2*^, YanLi Li^1*^*

**Figure S1. Nuclear‑cytoplasmic fractionation validation and ZNF609 mRNA expression following circ_0615 manipulation in EOC cells.**

**(A)** Nuclear-cytoplasmic separation detection of circ_0615 subcellular localization in Hey and Skov3 cell lines. GAPDH is used as a cytoplasmic endogenous reference, and Lamin B1 is used as a nuclear endogenous reference.

**(B)** qRT-PCR quantification of endogenous ZNF609 mRNA levels in Hey and Skov3 cells upon circ_0615 overexpression.

**(C)** qRT-PCR quantification of endogenous ZNF609 mRNA levels in Hey and Skov3 cells following circ_0615 knockdown.

ns, *p* > 0.05. Three independent experiments were performed.

**Figure S2. Migratory-apoptotic phenotypes under circ_0615 manipulation in EOC cells and Ki-67 staining of human EOC tissues.**

**(A)** Wound-healing assay for migratory capacity of EOC cells with circ_0615 overexpression. Images captured at 0 h and 24 h. Scale bar = 300 μm.

**(B)** Wound-healing assay for migratory potential of circ_0615-knockdown EOC cells. Images acquired at 0 h and 24 h. Scale bar = 300 μm.

**(C)** Flow-cytometric detection of apoptotic status in EOC cells upon circ_0615 overexpression.

**(D)** Flow-cytometric assessment of apoptotic phenotypes following circ_0615 knockdown in EOC cells.

**(E)** H&E staining and Ki-67 IHC staining of subcutaneous xenograft tumor tissues derived from circ_NC/circ_0615-overexpressing Hey cells in nude mice. Representative images are shown at 200× and 400× magnification. IHC staining was performed on formalin-fixed paraffin-embedded tissue sections using standard DAB-based IHC protocols. Scale bar = 100 μm (200×) and 40 μm (400×).

**p* < 0.05, ***p* < 0.01, ****p* < 0.001. Three independent experiments were performed.

**Figure S3. Bioinformatic prediction of circ_0615 coding potential, endogenous ZNF609-250aa polypeptide detection, and screening-based characterization of circ_0615-interacting eIF2α in EOC cells.**

**(A)** The predicted ORF box and encoding potential of circ_0615 (hsa_ZNF609_0001615) from circBase and circBank databases, along with a schematic diagram of circ_0615 encoding ZNF609-250aa.

**(B)** qRT-PCR quantification of circ_0615 transcript abundance following transfection with circ_0615-Flag and circ_0615-mut constructs.

**(C)** qRT-PCR quantification of ZNF609 mRNA abundance upon transfection of circ_0615-Flag and circ_0615-mut constructs.

**(D)** Western blot profiling of endogenous ZNF609-250aa polypeptides in normal ovarian (NC) and epithelial ovarian cancer (EOC) tissue specimens using ZNF609-specific antibody.

**(E)** Western blot profiling of endogenous ZNF609-250aa polypeptides across multiple ovarian cell lines using ZNF609-specific antibody.

**(F)** Results of mass spectrometry analysis indicated the related ~35 kDa proteins bound to circ_0615.

**(G)** Western blot to verify the binding of eIF2α protein antibodies to magnetic beads in RIP.

**(H)** qRT-PCR validation of siRNA-mediated eIF2α knockdown efficiency and circ_0000615 expression levels.

**p* < 0.05, ***p* < 0.01, ****p* < 0.001, ns, *p* > 0.05. Three independent experiments were performed.

**Figure S4. PDX tissue histopathological staining and cellular expression analyses of HMGB1 and ALDH1A1 under ZNF609-250aa/circ_0615 manipulation in epithelial ovarian cancer (EOC).**

**(A)** Representative H&E staining of P1-generation xenograft tissues derived from PDX mouse models of EOC. Tumor fragments were passaged from P0-generation PDX tumors and collected before AAV treatment. H&E staining was performed on formalin-fixed paraffin-embedded sections to confirm retention of human EOC tumor histomorphology prior to AAV intervention. Representative images are shown at 200× and 400× magnification. Scale bar = 100 μm (200×) and 40 μm (400×).

**(B)** Western blot and qRT-PCR measurement of HMGB1 protein and transcript abundance in EOC cells overexpressing ZNF609-250aa-Flag.

**(C)** Western blot and qRT-PCR determination of HMGB1 protein and transcript abundance in EOC cells with circ_0615 overexpression.

**(D)** Western blot and qRT-PCR measurement of HMGB1 protein and transcript abundance in circ_0615-knockdown EOC cells.

**(E)** Transfection of ZNF609-250aa-Flag in circ_0615-sh EOC cell line and western blot analysis of HMGB1 protein levels.

**(F)** IHC staining for Flag-tagged ZNF609-250aa, HMGB1, and Ki-67 in P1-generation EOC PDX tumor tissues following AAV2/8-NC or AAV2/8-ZNF609-250aa treatment. IHC staining was performed on formalin-fixed paraffin-embedded tissue sections using standard DAB-based IHC protocols. Representative images are shown at 200× and 400× magnification. Scale bar = 100 μm (200×) and 40 μm (400×).

**(G)** qRT-PCR analysis of HMGB1 and ALDH1A1 RNA and protein levels following HMGB1 knockdown in ZNF609-250aa-Flag EOC cell lines.

**p* < 0.05, ***p* < 0.01, ****p* < 0.001, ns, *p* > 0.05. Three independent experiments were performed.

**Table S1. Descriptive characteristics of EOC samples**

| Characteristic | | Epithelial Ovarian Cancer (n=70) |
| --- | --- | --- |
| **Median age (range)** | | 53 (20-78) |
| **Average age (range)** | | 50.2 (20-78) |
| **Gender** | Male | 0 (0%) |
|  | Female | 70 (100%) |
| **FIGO Stage, n (%)** | Ⅰ | 0 (0%) |
|  | Ⅱ | 0 (0%) |
|  | Ⅲ | 26 (37.14%) |
|  | Ⅳ | 44 (62.86%) |
| **Pathological characteristics, n (%)** | Metastasis | 28 (40.0%) |
|  | Lymphatic metastasis | 28 (40.0%) |
|  | Lympho-vascular invasion (LVI) | 28 (40.0%) |
|  | Neural invasion | 10 (14.29%) |
|  | Recurrence | 32 (45.71%) |
| **Histological subtype, n (%)** | High-grade serous carcinoma | 64 (91.4%) |
|  | Non-high-grade serous carcinoma | 6 (8.6%) |
| **Specimen origin,**  **n (%)** | Primary tumor | 70 (100%) |

**FIGO, International Federation of Gynecology and Obstetrics.**

**Table S2. Primer & shRNA & siRNA & Probe sequences**

| Sequence Name | Sequences（5'-3'） | Annealing temperature (°C) |
| --- | --- | --- |
| 18S RNA qRT-PCR primer | F: AGGAATTCCCAGTAAGTGCG | 55.0 |
|  | R: GCCTCACTAAACCATCCAA |  |
| U6 snRNA qRT-PCR primer | F: CTCGCTTCGGCAGCACA | 57.0 |
|  | R: AACGCTTCACGAATTTGCGT |  |
| GAPDH qRT-PCR primer | F: GACCCCTTCATTGACCTCAACTACATG | 60.0 |
|  | R: GTCCACCACCCTGTTGCTGTAGCC |  |
| circ_0615 qRT-PCR primer | F: AAGTCTGAAAAGCAATGATGTTG | 56.0 |
|  | R: CATCCCCACTGTCGTATGTCTC |  |
| ZNF609 qRT-PCR primer | F: AGATCATGGTTCGTACCCGATC | 59.0 |
|  | R: GCCTTCAAGGTTGACGCTAGTT |  |
| HMGB1 qRT-PCR primer | F: TATGGCAAAAGCGGACAAGG | 59.0 |
|  | R: CTTCGCAACATCACCAATGGA |  |
| ALDH1A1 qRT-PCR primer | F: GCACGCCAGACTTACCTGTC | 59.0 |
|  | R: CCTCCTCAGTTGCAGGATTAAAG |  |
| ChIP-ALDH1A1 qRT-PCR primer | F: AGGGACAGTTAGTTACATTTT | 50.0 |
|  | R: TTCTCAGATTTTTTTACTAAG |  |
| circ_0615 divergent primer | F: GCTCAATCCTTTGGGAACTA | 55.0 |
|  | R: GCCCCCAGCTTTCCTATTTT |  |
| circ_0615 convergent primer | F: AGGAGTGGATGCAAACCCG | 60.0 |
|  | R: GGGGTCACAAACTTGATGTTGTC |  |
| circ_0615-sh1 | F: GGGGCAGCGCTCAATCAATGA | N/A |
|  | R: TCATTGATTGAGCGCTGCCCC |  |
| circ_0615-sh2 | F: AGCGCTCAATCAATGATGTTG | N/A |
|  | R: CAACATCATTGATTGAGCGCT |  |
| si-NC | F: UUCUCCGAACGUGUCACGUTT | N/A |
|  | R: ACGUGACACGUUCGGAGAATT |  |

**Table S2. (continued)**

| Sequence Name | Sequences（5'-3'） | Annealing temperature (°C) |
| --- | --- | --- |
| si-eIF2α-1 | F: GGGUGUGUUCAAUGUUCAAAU | N/A |
|  | R: UUGAACAUUGAACACACCCCU |  |
| si-eIF2α-2 | F: CAUUAAGAUUAAUCUAAUAGC | N/A |
|  | R: UAUUAGAUUAAUCUUAAUGGG |  |
| si-eIF2α-3 | F: GAUUCUUCUUAGUGAAUUAUC | N/A |
|  | R: UAAUUCACUAAGAAGAAUCAU |  |
| si-HMGB1-1 | F: GGGAGGAGCAUAAGAAGAATT | N/A |
|  | R: UUCUUCUUAUGCUCCUCCCTT |  |
| si-HMGB1-2 | F: GGAGAGAUGUGGAAUAACATT | N/A |
|  | R: UGUUAUUCCACAUCUCUCCTT |  |
| si-HMGB1-3 | F: GGAAGACCAUGUCUGCUAATT | N/A |
|  | R: UUAGCAGACAUGGUCUUCCTT |  |
| RNA-pull-down NC-probe | ACACUGUCGAAUCGCUAUAUUCUAU | N/A |
| RNA-pull-down circ_0615-probe | AAGCAAUGAUGUUGUCCACUGGGGC | N/A |
| FISH circ_0615-probe | CAGCGCTCAATCCTTCAATGATGTT | N/A |

**Annealing temperature, the temperature set for the annealing step of qRT-PCR. N/A indicates not applicable.**

**Table S3. LC-MS/MS identification results of the ~34 kDa gel-excised protein band**

| Name | Mean |
| --- | --- |
| PG.UniProtIds | O15014 (ZN609_HUMAN) |
| PG.MolecularWeight (Da) | 25397.22 |
| PG.Coverage (%) | 9.64 |
| PG.UniquePeptides | 2 |
| PG.PSMs | 15 |
| PG.Qvalue | < 0.0001 |
| PEP.StrippedSequence | SGDGANAGGLVAAIAPKGSEKAAK |
